# Evaluation of Promoter Methylation of *RASSF1A* and *ATM* in Peripheral Blood of Breast Cancer Patients and Healthy Control Individuals

**DOI:** 10.3390/ijms19030900

**Published:** 2018-03-19

**Authors:** Xue Cao, Qiuqiong Tang, Tim Holland-Letz, Melanie Gündert, Katarina Cuk, Sarah Schott, Jörg Heil, Michael Golatta, Christof Sohn, Andreas Schneeweiss, Barbara Burwinkel

**Affiliations:** 1 Molecular Biology of Breast Cancer, Department of Gynecology and Obstetrics, University of Heidelberg, Heidelberg 69120, Germany; tangqiuqiong2017@foxmail.com (Q.T.); melanie.guendert@t-online.de (M.G.); k.cuk@Dkfz-Heidelberg.de (K.C.); Sarah.Schott@med.uni-heidelberg.de (S.S.); Christof.Sohn@med.uni-heidelberg.de (C.S.); Andreas.Schneeweiss@med.uni-heidelberg.de (A.S.); 2 Division of Molecular Epidemiology (C080), German Cancer Research Center (DKFZ), Heidelberg 69120, Germany; 3 Division of Biostatistics (C060), German Cancer Research Center (DKFZ), Heidelberg 69120, Germany; t.holland-letz@Dkfz-Heidelberg.de; 4 Department of Gynecology and Obstetrics, University Women’s Clinic, Heidelberg 69120, Germany Joerg.Heil@med.uni-heidelberg.de (J.H.); Michael.Golatta@med.uni-heidelberg.de (M.G.); 5 National Centre for Tumor Diseases, Heidelberg 69120, Germany

**Keywords:** breast cancer, DNA methylation, *RASSF1A*, *ATM*

## Abstract

Breast cancer (BC) is the most common cancer among women and has high mortality rates. Early detection is supposed to be critical for the patient’s prognosis. In recent years, several studies have investigated global DNA methylation profiles and gene-specific DNA methylation in blood-based DNA to develop putative screening markers for cancer. However, most of the studies have not yet been validated. In our study, we analyzed the promoter methylation of *RASSF1A* and *ATM* in peripheral blood DNA of 229 sporadic patients and 151 healthy controls by the MassARRAY EpiTYPER assay. There were no significant differences in DNA methylation levels of *RASSF1A* and *ATM* between the sporadic BC cases and the healthy controls. Furthermore, we performed the Infinium HumanMethylation450 BeadChip (450K) array analysis using 48 sporadic BC cases and 48 healthy controls (cases and controls are the same from those of the MassARRAY EpiTYPER assay) and made a comparison with the published data. No significant differences were presented in DNA methylation levels of *RASSF1A* and *ATM* between the sporadic BC cases and the healthy controls. So far, the evidence for powerful blood-based methylation markers is still limited and the identified markers need to be further validated.

## 1. Introduction

Breast cancer (BC) is one of the most common cancers in women worldwide [1,2]. The early detection of breast cancer plays an important role in successful treatment and outcome. To date, mammography is still the main screening method for breast cancer early detection. However, the limitations of this method are noticed as tumors can only be partially identified in women (mostly young women) with dense breasts [3,4]. Therefore, the identification of new reliable biomarkers for the screening and diagnosis of breast cancer is urgently needed.

Epigenetic events are critical factors in the development of human cancers [5,6,7,8]. Aberrant methylation in the promoter regions of tumor suppressor genes is related to carcinogenesis through transcriptional silencing of gene expression [9], leading to the initiation and progression of cancer [10,11]. Global hypomethylation and gene-specific hypermethylation were shown to be associated with malignancy [12,13,14]. Several studies showed that these epigenetic changes were early events of a variety of cancers including breast [15,16,17], lung [18], and colon cancer [19], and all of them could be recognized as common hallmarks in different kinds of tumors [20]. Similar alterations also existed in blood-derived DNA, suggesting that blood-based DNA methylation could reveal new biomarkers for BC screening or diagnosis [21,22]. A major advantage of blood-based DNA methylation is the easy accessibility of blood samples to investigate DNA methylation in cancer patients.

Recently, studies have been focused on two tumor suppressor genes, RAS-association domain family member 1A (*RASSF1A*) and ataxia-telangiectasia mutated gene (*ATM*). *RASSF1A* takes part in apoptosis induction, proliferation regulation, and microtubules stabilization [23]. Aberrant hypermethylation of *RASSF1A* has been demonstrated in various solid tumors, including lung, prostate, ovary, and breast cancer tissue samples [24,25,26,27]. Furthermore, a previous study has demonstrated no *RASSF1A* promoter hypermethylation in peripheral blood from normal blood donors, which suggested the potential of *RASSF1A* as a biomarker for cancers [28]. *ATM* plays a critical role in repairing DNA double-strand breaks and is involved in numerous processes including recognition of damaged DNA, recruitment of repair proteins, signaling to cell cycle checkpoints, transcriptional regulation, and activation of apoptosis [29]. Hypermethylation of the *ATM* promoter has been shown in gastric lymphoma, glioma, colonic cancer, adenoma, and breast cancer tissue samples [30,31,32,33]. Moreover, we summarized recent studies which investigated promoter methylation changes of *RASSF1A* and *ATM* in peripheral blood (plasma, serum, or whole blood) from BC patients and healthy controls (see Supplementary Table S1). To our surprise, the results of these studies were inconsistent. The promoter methylation of *RASSF1A* did not show a significant difference between BC patients and healthy controls in two quantitative studies [34,35]. It is worth noting that the sample sizes analyzed in these two studies were rather low. For *ATM*, two studies reported its hypermethylation in BC patients, but the CpG sites they investigated were located in the gene body or intragenic region instead of the promoter [36,37]. Altogether, blood-based DNA methylation of the *RASSF1A* and *ATM* promoter remains unclear in BC patients. However, these findings reveal the potential of *RASSF1A* and *ATM* promoter hypermethylation as novel biomarkers for cancer early detection.

Therefore, in this study, we aimed to conduct a case-control study with a large sample size to investigate *RASSF1A* and *ATM* promoter methylation in BC patients and healthy controls. A quantitative method, the MassARRAY EpiTYPER assay, was applied by using peripheral blood DNA in order to find useful blood-based biomarkers for BC early detection.

In addition, we compared previously published results of blood-based DNA methylation of specific genes found to be associated with breast cancer with our results by Infinium HumanMethylation450 BeadChip (450K) array analysis of sporadic breast cancer cases and healthy controls.

## 2. Results

### 2.1. Promoter Methylation Levels of RASSF1A and ATM in BC Patients and Healthy Controls and Its Correlation to Clinical Characteristics

In order to investigate the promoter methylation levels of *RASSF1A* and *ATM*, we performed the MassARRAY EpiTYPER assay by using peripheral blood DNA from BC patients and healthy controls. Our results showed that the promoter methylation levels of *RASSF1A* and *ATM* were quite low in both BC patients and healthy controls. In the amplicon of *RASSF1A*, 17 CpG sites were measured and the average methylation levels of all these CpG sites were 0.037 and 0.042 in the BC patients and the healthy controls, respectively. Compared to the healthy controls, no significant difference was represented in the methylation levels of the BC patients among all these 17 CpG sites (see Table 1).

As for *ATM*, 30 CpG sites were analyzed in its amplicon. The average methylation levels of all investigated CpG sites of *ATM* were 0.047 and 0.048 in the BC patients and the healthy controls, respectively. No visible difference was detected in the methylation levels of these CpG sites between the BC cases and the healthy controls (see Table 2). The CpG sites of *RASSF1A* and *ATM* analyzed in this study are shown in Supplementary Figure S1.

To further explore the association between the promoter methylation of *RASSF1A*/*ATM* and the clinical characteristics of the BC patients, statistical analyses were performed. All the BC patients were classified into different subgroups according to their clinical properties (see Table 3). To our surprise, no significant correlation was represented between the methylation levels of all the CpG sites in *RASSF1A* or *ATM* amplicons and the clinical characteristics of the BC patients (see Supplementary Tables S2 and S3).

Taken together, these results demonstrated that the promoter methylation levels of *RASSF1A* and *ATM* included in our study were not statistically different between the BC patients and the healthy controls.

### 2.2. Comparison of the Results from This Study with the Results of Infinium HumanMethylation450 BeadChip Array and with Literature

To better interpret the results of the Sequenom MassARRAY EpiTYPER assay, we also performed an epigenome-wide Infinium HumanMethylation450 BeadChip array (450K array). In line with the MassARRAY data, the methylation levels of all the investigated CpG sites of *RASSF1A* and *ATM* were rather low. No significant difference was revealed in the methylation levels of *RASSF1A* and *ATM* between the BC patients and the healthy controls. Interestingly, we identified four CpG sites of *RASSF1A* and 13 CpG sites of *ATM* which were identical in both the Sequenom MassArray EpiTYPER assay and the 450K array. These data confirmed the findings of our MassARRAY analysis (see Table 4).

To understand the current research status on gene-specific DNA methylation in blood-based DNA as a screening biomarker for breast cancer early detection, we reviewed recent studies which investigated blood-based DNA methylation of *BRCA1*, *APC*, *RARB*, *ESR1*, *CDH1*, *SYK*, *TIMP3*, *GSTP1*, *DAPK*, and *IGF2* between BC patients and healthy controls [38]. Interestingly, the results of blood DNA methylation levels of *BRCA1*, *APC*, *RARB*, *ESR1*, *TIMP3*, and *GSTP1* were inconsistent among the previous studies, but a higher frequency of methylated *DAPK* in peripheral DNA was reported in BC cases compared to healthy controls in several studies (see Supplementary Table S4) [36,37,39,40]. Moreover, the methylation levels of *CDH1*, *IGF2*, and *SYK* were not significantly different in peripheral blood DNA between BC cases and healthy controls according to the published data [34,35,41,42,43,44].

Next, we investigated the methylation levels of these genes by a 450K array from our own group. Compared to the healthy controls, the methylation levels did not show a significant difference in most of the CpG sites of these genes in the BC cases, except for a few CpG sites including *BRCA1* (cg13782816), *APC* (cg01240931 and cg14511739), *ESR1* (cg25565730), *CDH1* (cg26508465), and *GSTP1* (cg06841499) (see Supplementary Table S4).

## 3. Discussion

In this work, we conducted a large cohort case-control study to investigate the promoter methylation changes of two tumor suppressor genes (*RASSF1A* and *ATM*) in peripheral blood DNA as potential epigenetic markers for breast cancer risk and early detection. To our surprise, no significant differences in the methylation level of *RASSF1A* and *ATM* in peripheral blood DNA were revealed between BC cases and healthy controls.

*RASSF1A* methylation in BC patients has been analyzed by several groups using either blood serum or blood plasma samples [39,40,45,46,47,48,49,50]. Jo-Heon Kim et al. observed significantly higher methylation frequencies of *RASSF1A* in ductal carcinoma in situ (DCIS) or invasive ductal carcinoma (IDC) than in control subjects, but the CpG sites they investigated in the *RASSF1A* promoter region were different from ours [49]. In contrast, Zmetakova et al., evaluated DNA methylation profiles of the *RASSF1A* promoter by pyrosequencing in invasive breast cancer. They demonstrated no significant difference in peripheral blood DNA between BC cases and healthy controls, which was in line with our findings [35]. Moreover, another study by Brooks et al., also showed no significant difference in *RASSF1A* promoter methylation between BC cases and controls, although they used serum instead of whole blood samples. Interestingly, the CpG sites analyzed in their study partially overlapped with some CpG sites in our *RASSF1A* amplicon [51].

For *ATM*, we demonstrated that the promoter methylation level in peripheral blood DNA of BC patients was not significantly different from that of healthy controls. Consistent with this, Flanagan et al. detected no significant difference in methylation of the *ATM* promoter CpG islands in white blood cell DNA between BC cases and healthy controls [36].

In fact, it is difficult to compare the results between our study and the published data, where different techniques for methylation analysis have been used and different CpG sites have been investigated. Moreover, depending on the technique used for quantification, methylation levels can differ, even for the same CpG site [52]. The promoter region is located at around 100–1000 base pairs before the transcription start sites. A length of around 100–500 base pairs can be amplified by PCR, which means that the investigation cannot cover the entire promoter region. Currently, there is no standard for blood DNA methylation analysis. The exact quantity of methylated CpG sites as the biomarker for breast cancer risk remains unclear.

In our study, we chose whole blood DNA to investigate the methylation levels. Recently, the pivotal significance of circulating tumor cells (CTCs) has been realized for breast cancer patients, even in patients without metastases. It is possible that the whole blood was mixed by CTCs, further affecting the analysis of *RASSF1A* or *ATM* promoter methylation. In fact, peripheral blood samples from epidemiological studies always comprise mixed cell populations, but it is not feasible or practical to fractionate cell populations in an epidemiological study setting [53]. At present, many studies use cell-free DNA (cfDNA) to investigate DNA methylation, but its low concentration and small fragments may disrupt the detection of blood-based DNA methylation [54,55,56]. Even if the pooling method can improve the concentration of cfDNA, the possible bias may still occur. In addition, the origin of cfDNA is still uncertain and it requires more evidence to elucidate the mechanism of cfDNA release [57].

Radpour et al. demonstrated that heterogeneity of methylation changes exists in carcinogenesis, but no single gene has been shown to be methylated in all types of breast cancer [58]. Therefore, a panel of genes should be considered as biomarkers for breast cancer screening. Our 450K data analyzed additional ten genes to screen for potential methylation CpG sites as biomarkers for breast cancer early detection. The methylation levels did not show a significant difference in most of the CpG sites of these genes in BC cases.

With the analysis of *RASSF1A* and *ATM* using the MassARRAY EpiTYPER assay, we could confirm the results of our 450K methylation data for these two genes. However, the methylation levels detected in our samples were quite low. We identified that the average methylation levels of *RASSF1A* and *ATM* were at around 0.04 and 0.05, respectively. In accordance with this, a study by Cho et al., reported that the methylation level of *RASSF1A* was below 4% [34]. The MassARRAY system could detect the methylation level as low as 5% [59]. For some of the analyzed CpG sites, no detectable quantitative value could be gained because the results were below this detection limit. Future studies applying digital PCR techniques in next-generation sequencing (PCR/NGS)-based analysis may improve the detection limit.

Another limitation of the 450K results was the limited sample size, which included only 48 cases and 48 controls. Thus, larger multicenter prospective study cohorts are needed to validate these results.

In conclusion, the promoter CpG methylation status of *RASSF1A* and *ATM* in peripheral blood included in our study was unable to distinguish between BC cases and healthy controls. Further prospective studies should be carried out to evaluate whether *RASSF1A* or *ATM* promoter methylation could be suitable biomarkers for breast cancer early detection [39].

## 4. Materials and Methods

### 4.1. Study Population

This study was approved by the Ethics Committee of University Hospital in Heidelberg (S-039/2008, 27 April 2009; S-175/2010, 26 May 2010). All samples of the BC cases and the healthy controls were obtained from centers in southwest Germany. All the enrolled patients and the healthy controls were Caucasian and were given written informed consent. Peripheral whole-blood samples from the BC patients were successively collected before therapeutic treatments at the University Hospital of Heidelberg. Clinical characteristics of the BC patients were defined according to the American Joint Committee on Cancer staging manual [60]. Detailed characteristics of the sporadic BC cases were described in Table 3. In the group of healthy controls, peripheral whole-blood samples were consecutively collected from blood donors at the University Hospital of Heidelberg. Donors approved the use of their blood samples for research purposes. All the donors were healthy when donating blood and none of them had a family history of BC. Blood samples were collected between 2011 and 2014 and a total of 229 sporadic BC patients and 151 healthy controls were randomly selected for this study (see Table 5).

### 4.2. DNA Isolation and Bisulfite Conversion

DNA was isolated from 200 µL aliquots of whole blood using the QIAamp DNA Blood Mini Kit (Qiagen, Hilden, Germany) according to the manufacturer’s recommendations. NanoDrop ND-1000 UV/Vis-Spectralphotometer 3.3 (peqLab, Erlangen, Germany) was used to measure DNA quality and quantity. DNA bisulfite treatment was carried out using the EZ-96 DNA methylation Gold kit (Zymo Research Corporation, Orange, CA, USA) as described by the manufacturer.

### 4.3. Primer Design and PCR Amplification

The PCR primers for *RASSF1A* and *ATM* amplicon sequences (see Table 6 and Table 7) were designed with the online tool “epidesigner” (http://www.epidesigner.com/start3.html). PCR was performed in a final reaction volume of 6 µL which included bisulfite-treated DNA (10 ng/µL), CoralLoad Buffer (10×; Qiagen, Valencia, CA, USA), forward and reverse primers (1 µM of each; Sigma, Darmstadt, Germany), dNTPs (10 mM), and HotStar Taq DNA polymerase (5 U/µL; Qiagen, Valencia, CA, USA). The touch-down PCR profile was 5 min of activation at 95 °C, 30 s of denaturation at 94 °C, and 30 s of annealing at a temperature reduced from 59 °C to 53 °C (every 2 °C), followed by a final extension at 72 °C for 1 min. After a maintenance of 5 min at 72 °C, the reaction ended up at 4 °C. PCR products were electrophoresed on 1% agarose gels and evaluated under ultraviolet light.

### 4.4. Methylation Analysis

For methylation analysis, the Sequenom MassARRAY EpiTYPER assay was applied as described previously [61]. The PCR amplicons were conducted subsequently according to the protocol of the Sequenom EpiTYPER Assay and cleaned by Resin. A nanodispenser was used to transfer the products to a 384 SpectroCHIP (SEQUENOM, San Diego, CA, USA). The chips were read by a Sequenom Mass Spectrometer system (SEQUENOM, San Diego, CA, USA). Data was gathered by SpectroACQUIRE v3.3.1.3 software (SEQUENOM, San Diego, CA, USA) and visualized with MassArray EpiTYPER v1.0 software (SEQUENOM, San Diego, CA, USA). Results were depicted as “beta” values (β) between 0 and 1.

### 4.5. 450K Methylation Study

Epigenome-wide DNA methylation profiling on 96 age matched blood DNAs from 48 sporadic BC cases and 48 healthy controls was performed by applying Infinium HumanMethylation450 BeadChip (450K) as described before [61]. In brief, DNA was extracted from whole blood samples. Then, the DNA samples were bisulfate converted, purified, and applied to the BeadChips (Illumina, San Diego, CA, USA). Image processing and data extraction were performed following Illumina’s instructions. Further details of the 450K array analysis were given in Tang et al. [61].

### 4.6. Statistical Analysis

SPSS statistics 24.0 (IBM, NY, USA) was used for statistical analyses of the data. Normality of distribution was evaluated by the Kolmogorov-Smirnoff test. Non-normally distributed data was analyzed by the nonparametric Mann-Whitney *U* or Kruskal-Wallis *H* test. All tests were performed two-tailed at the significance level *p* < 0.05.

## Figures and Tables

**Table 1:** Comparison of *RASSF1A* DNA methylation between the BC patients and the healthy controls in peripheral blood.

CpG Site	Case, *n*	Control, *n*	BC Cases Median (IQR)	Controls Median (IQR)	*p*-Value *	*p*-Value **
*RASSF1A*_CpG_1	222	144	0.00 (0.00–0.00)	0.00 (0.00–0.00)	0.03	0.31
*RASSF1A*_CpG_8	216	134	0.00 (0.00–0.02)	0.00 (0.00–0.02)	1.00	0.76
*RASSF1A*_CpG_9	223	141	0.01 (0.00–0.02)	0.01 (0.00–0.03)	1.00	0.78
*RASSF1A*_CpG_11,12	187	121	0.13 (0.11–0.16)	0.14 (0.11–0.17)	1.00	0.38
*RASSF1A*_CpG_13	222	141	0.04 (0.00–0.08)	0.04 (0.00–0.08)	1.00	0.90
*RASSF1A*_CpG_14,15	187	121	0.13 (0.11–0.16)	0.14 (0.11–0.17)	1.00	0.35
*RASSF1A*_CpG_16	226	146	0.02 (0.00–0.03)	0.02 (0.02–0.03)	1.00	0.15
*RASSF1A*_CpG_19	216	134	0.00 (0.00–0.02)	0.00 (0.00–0.02)	1.00	0.09
*RASSF1A*_CpG_20	216	134	0.00 (0.00–0.02)	0.00 (0.00–0.02)	1.00	0.34
*RASSF1A*_CpG_21,22	226	145	0.05 (0.04–0.06)	0.05 (0.03–0.07)	0.62	0.64
*RASSF1A*_CpG_23	226	146	0.00 (0.00–0.00)	0.00 (0.00–0.00)	0.28	0.70
*RASSF1A*_CpG_24	226	145	0.03 (0.02–0.05)	0.03 (0.02–0.07)	1.00	0.79
*RASSF1A*_CpG_25	223	141	0.01 (0.00–0.02)	0.01 (0.00–0.03)	1.00	0.31
*RASSF1A*_CpG_26	226	146	0.04 (0.03–0.05)	0.04 (0.03–0.05)	1.00	0.76
MEAN	226	146	0.037 (0.029–0.048)	0.042 (0.032–0.054)	0.25	0.78

Abbreviations: IQR: interquartile range. * *p*-value for the difference between controls and patients was analyzed by Mann-Whitney *U* test and was adjusted by Bonferroni-Holm method, α = 0.00333. ** *p*-value for the difference between controls and patients was analyzed by logistic regression and was adjusted by age, α = 0.05.

**Table 2:** Comparison of *ATM* DNA methylation between the BC patients and the healthy controls in peripheral blood.

CpG Site	Case, *n*	Control, *n*	BC Cases Median (IQR)	Controls Median (IQR)	*p*-Value *	*p*-Value **
*ATM*_CpG_1	222	146	0.02 (0.01–0.03)	0.02 (0.01–0.02)	1.00	0.95
*ATM*_CpG_2,3,4,5	142	95	0.07 (0.06–0.10)	0.07 (0.06–0.09)	1.00	0.91
*ATM*_CpG_6	223	146	0.00 (0.00–0.00)	0.00 (0.00–0.00)	1.00	0.37
*ATM*_CpG_7,8	223	146	0.05 (0.03–0.06)	0.04 (0.03–0.06)	1.00	0.60
*ATM*_CpG_10,11	221	145	0.11 (0.10–0.13)	0.12 (0.10–0.13)	1.00	0.95
*ATM*_CpG_12	223	146	0.00 (0.00–0.00)	0.00 (0.00–0.00)	1.00	0.94
*ATM*_CpG_13,14	223	146	0.03 (0.02–0.04)	0.03 (0.02–0.04)	1.00	0.43
*ATM*_CpG_17	219	145	0.00 (0.00–0.04)	0.01 (0.00–0.04)	1.00	0.73
*ATM*_CpG_18,19	223	146	0.13 (0.12–0.16)	0.14 (0.12–0.18)	0.22	0.18
*ATM*_CpG_20,21	223	146	0.02 (0.02–0.03)	0.02 (0.02–0.03)	1.00	0.36
*ATM*_CpG_26	219	146	0.06 (0.04–0.09)	0.07 (0.05–0.09)	1.00	0.21
*ATM*_CpG_27	223	146	0.11 (0.10–0.12)	0.11 (0.10–0.12)	1.00	0.66
*ATM*_CpG_28	222	146	0.02 (0.02–0.03)	0.02 (0.02–0.03)	1.00	0.75
*ATM*_CpG_29	223	146	0.03 (0.02–0.03)	0.03 (0.02–0.03)	1.00	0.68
*ATM*_CpG_32	223	145	0.00 (0.00–0.00)	0.00 (0.00–0.00)	1.00	0.53
*ATM*_CpG_33	223	146	0.00 (0.00–0.00)	0.00 (0.00–0.00)	1.00	0.15
*ATM*_CpG_34	223	146	0.13 (0.10–0.16)	0.14 (0.10–0.18)	1.00	0.70
*ATM*_CpG_35	219	145	0.00 (0.00–0.04)	0.01 (0.00–0.04)	1.00	0.78
*ATM*_CpG_36	223	145	0.00 (0.00–0.00)	0.00 (0.00–0.00)	1.00	0.14
*ATM*_CpG_37	223	146	0.13 (0.10–0.16)	0.14 (0.10–0.18)	1.00	0.16
*ATM*_CpG_38	192	135	0.00 (0.00–0.01)	0.00 (0.00–0.01)	1.00	0.95
*ATM*_CpG_39	223	146	0.02 (0.01–0.04)	0.02 (0.01–0.03)	1.00	0.91
MEAN	223	146	0.047 (0.042–0.055)	0.048 (0.042–0.056)	1.00	0.37

Abbreviations: IQR: interquartile range. * *p*-value for the difference between controls and patients was analyzed by Mann-Whitney *U* test and was adjusted by Bonferroni-Holm method, α = 0.00227. ** *p*-value for the difference between controls and patients was analyzed by logistic regression and was adjusted by age, α = 0.05.

**Table 3:** Characteristics of the sporadic BC patients.

Characteristics	BC Patients Number (%)
Tumor, lymph node and metastasis (TNM) Stage	
stage 0	1 (0.4%)
stage I	69 (30.1%)
stage II	72 (31.4%)
stage III	15 (6.6%)
stage IV	4 (1.7%)
neoadjuvant chemotherapy *	50 (21.8%)
unknown	18 (7.9%)
Type of BC	
Ductal	179 (78.2%)
Lobular	13 (5.7%)
Ductal-Lobular	3 (1.3%)
ductal carcinoma in situ (DCIS)	4 (1.7%)
Others	10 (4.4%)
unknown	24 (10.5%)
Estrogen receptor (ER) Status ^a^	
negative	21 (9.2%)
positive	160 (69.9%)
unknown	48 (21.0%)
Progesterone receptor (PR) Status ^a^	
negative	36 (15.7%)
positive	145 (63.3%)
unknown	48 (21.0%)
Human epidermal growth factor receptor 2 (HER2) Status ^b^	
negative	165 (72.1%)
positive	16 (7.0%)
unknown	48 (21.0%)

^a^ Immunoreactive score (IRS): ER/PR negative: IRS 0–2; ER/PR positive: IRS 3–12. ^b^ HER2 negative: IHC-score 0–1; HER2 positive: IHC-score 3; If IHC-score = 2, FISH/CISH was further analyzed, HER2 is recognized as positive if it is amplified. * Patients were treated with neoadjuvant chemotherapy, no stage is given here.

**Table 4:** Comparison of methylation levels of peripheral blood DNA between different analytical methods.

Gene	450K Results					Sequenom MassARRAY EpiTYPER Assay				
	CpG	Cases No./CTL No.	BC Cases Mean ± SD	CTL Mean ± SD	*p*-Value ^a^	CpG	Cases No./CTL No.	BC Cases Median (IQR)	CTL Median (IQR)	*p*-Value ^b^
***RASSF1A***	cg 12966367	48/48	0.029 ± 0.005	0.027 ± 0.004	0.19	CpG_1	229/151	0.00 (0.00–0.00)	0.00 (0.00–0.00)	0.03
	cg25486143	48/48	0.014 ± 0.002	0.014 ± 0.002	0.80	CpG_14	229/151	0.13 (0.11–0.16)	0.14 (0.11–0.17)	1.00
	cg06172942	48/48	0.038 ± 0.007	0.038 ± 0.011	0.91	CpG_15	229/151	0.13 (0.11–0.16)	0.14 (0.11–0.17)	1.00
	cg 03297783	48/48	0.015 ± 0.002	0.016 ± 0.002	0.35	CpG_19	229/151	0.00 (0.00–0.02)	0.00 (0.00–0.02)	1.00
	cg08047457 *	48/48	0.030 ± 0.004	0.030 ± 0.004	0.87	CpG_2	229/151	—	—	—
	cg25747192 *	48/48	0.043 ± 0.007	0.043 ± 0.008	0.99	CpG_3	229/151	—	—	—
	cg21554552 *	48/48	0.031 ± 0.005	0.030 ± 0.005	0.42	CpG_4	229/151	—	—	—
	cg27569446 *	48/48	0.012 ± 0.002	0.011 ± 0.002	0.65	CpG_5	229/151	—	—	—
	cg04540383 *	48/48	0.032 ± 0.004	0.033 ± 0.005	0.92	CpG_18	229/151	—	—	—
	Mean	48/48	0.204 ± 0.005	0.205 ± 0.005	0.56	Mean	229/151	0.037 (0.029–0.048)	0.042 (0.032–0.054)	0.25
***ATM***	cg19288979	48/48	0.075 ± 0.006	0.076 ± 0.007	0.95	CpG_1	229/151	0.02 (0.01–0.03)	0.02 (0.01–0.02)	1.00
	cg10610482	48/48	0.040 ± 0.005	0.037 ± 0.005	0.31	CpG_2	229/151	0.07 (0.06–0.10)	0.07 (0.06–0.09)	1.00
	cg12848864	48/48	0.051 ± 0.007	0.048 ± 0.007	0.38	CpG_4	229/151	0.07 (0.06–0.10)	0.07 (0.06–0.09)	1.00
	cg03165700	48/48	0.047 ± 0.007	0.044 ± 0.005	0.16	CpG_5	229/151	0.07 (0.06–0.10)	0.07 (0.06–0.09)	1.00
	cg15504467	48/48	0.027 ± 0.003	0.026 ± 0.004	0.36	CpG_6	229/151	0.00 (0.00–0.00)	0.00 (0.00–0.00)	1.00
	cg05033322	48/48	0.028 ± 0.004	0.027 ± 0.003	0.82	CpG_7	229/151	0.05 (0.03–0.06)	0.04 (0.03–0.06)	1.00
	cg16693212	48/48	0.033 ± 0.004	0.031 ± 0.004	0.29	CpG_8	229/151	0.05 (0.03–0.06)	0.04 (0.03–0.06)	1.00
	cg15370815	48/48	0.074 ± 0.007	0.072 ± 0.007	0.60	CpG_10	229/151	0.11 (0.10–0.13)	0.12 (0.10–0.13)	1.00
	cg16788234	48/48	0.052 ± 0.008	0.051 ± 0.007	0.82	CpG_11	229/151	0.11 (0.10–0.13)	0.12 (0.10–0.13)	1.00
	cg24030675	48/48	0.013 ± 0.001	0.013 ± 0.001	0.58	CpG_17	229/151	0.00 (0.00–0.04)	0.01 (0.00–0.04)	1.00
	cg06053805	48/48	0.012 ± 0.001	0.011 ± 0.003	0.14	CpG_18	229/151	0.13 (0.12–0.16)	0.14 (0.12–0.18)	0.22
	cg06750635	48/48	0.012 ± 0.001	0.011 ± 0.001	0.69	CpG_19	229/151	0.13 (0.12–0.16)	0.14 (0.12–0.18)	0.22
	cg25400013	48/48	0.016 ± 0.002	0.016 ± 0.001	0.94	CpG_21	229/151	0.02 (0.02–0.03)	0.02 (0.02–0.03)	1.00
	cg20342375 *	48/48	0.021 ± 0.003	0.020 ± 0.002	0.085	CpG_9	229/151	—	—	—
	cg22837512 *	48/48	0.023 ± 0.003	0.023 ± 0.002	0.99	CpG_15	229/151	—	—	—
	cg18391757 *	48/48	0.025 ± 0.004	0.025 ± 0.004	0.72	CpG_22	229/151	—	—	—
	Mean	48/48	0.236 ± 0.002	0.235 ± 0.002	0.79	Mean	229/151	0.047 (0.042–0.055)	0.048 (0.042–0.056)	1.00

^a^ *p*-value was ajusted by age, batch, cell type and multiple test. ^b^ *p*-value for the difference between breast cancer patients and healthy controls was analyzed by Mann-Whitney *U* test and was adjusted by Bonferroni-Holm method, α = 0.00333 and α = 0.00227 for *RASSF1A* and *ATM* respectively. * CpG sites were included in *RASSF1A* and *ATM* amplicons but were not investigated by Sequenom MassARRAY EpiTYPER assay in this study. Abbreviations: CTL, control; BC, breast cancer.

**Table 5:** Sample Information.

Gene	Sample Type	Group	Number	Age (*y*, Mean ± SD)
*RASSF1A*	Peripheral blood DNA	Sporadic BC	229	48.37 ± 7.08
		Controls	151	43.76 ± 14.49
*ATM*	Peripheral blood DNA	Sporadic BC	229	48.37 ± 7.08
		Controls	151	43.76 ± 14.49

**Table 6:** Bisulfite-specific primers for the target amplicons.

Target	Primer	Sequence (5′-3′)
***RASSF1A***	sense	aggaagagagGTAATGGAAATTTGGGTGTAGGGAT
	antisense	cagtaatacgactcactatagggagaaggctCTAACAACCCAAAATAACAAAACCA
***ATM***	sense	aggaagagagAGGGAAAATTTTTGGTTTTAAAGGT
	antisense	cagtaatacgactcactatagggagaaggctCCATATCCACCAATAACCAAC

**Table 7:** Sequences of the target amplicons.

Amplicon	Sequence (5′-3′)
***RASSF1A***	GCAATGGAAACCTGGGTGCAGGGACTGTGGGGCCCGAAGGCGGGGCTGGGCGCGCTCTCGCAGAGCCCCCCCCGCCTTGCCCTTCCTTCCCTCCTTCGTCCCCTCCTCACACCCCACCCCGGACGGCCACAACGACGGCGACCGCAAAGCACCACGCGGAGATACCCGTGTTTCTGGAGGCCAGCTTTACTGTGCTAGAGGAAGAGGGTCCCCACATCCGGCCCTGGCCCTCCTGGTCCGGTTTGCTGAAGCAACACACTTGGCCTACCCACTGGGTGGGGCAGGAAGTCTCGAGCCTTCACTTGGGGTGAGGAGGAGGGAGATCGGTCAGCAGCTTTACCGCCCGCTCTGCTCTCCACTGCGGAGACTGGGGCTCCGGCAGAGGCTGGACCGTGATCTTGAGGTTCAGGGGTGCATTCTGGGTGGATTCCCTTGGCATGGGTGGTCGGCCCTCAGCAACTGCAGCCCTCATTTGGCTCTGTCACCCTGGGCTGCCAG
***ATM***	AGGGAAAACCTTTGGCCTCAAAGGTCCTTCTGTCCAGCATAGCCGGGTCCAATAACCCTCCATCCCGCGTCCGCGCTTACCCAATACAAGCCGGGCTACGTCCGAGGGTAACAACATGATCAAAACCACAGCAGGAACCACAATAAGGAACAAGACTCAGGTTAAAGCAAACACAGCGACAGCTCCTGCGCCGCATCTCCTGGTTCCAGTGGCGGCACTGAACTCGCGGCAATTTGTCCCGCCTCTTTCGCTTCACGGCAGCCAATCGCTTCCGCCAGAGAAAGAAAGGCGCCGAAATGAAACCCGCCTCCGTTCGCCTTCGGAACTGTCGTCACTTCCGTCCTCAGACTTGGAGGGGCGGGGATGAGGAGGGCGGGGAGGACGACGAGGGCGAAGAGGGTGGGTGAGAGCCCCGGAGCCCGAGCCGAAGGGCGAGCCGCAAACGCTAAGTCGCTGGCCATTGGTGGACATGG

## Supplementary Materials

Supplementary materials can be found at http://www.mdpi.com/1422-0067/19/3/900/s1.

## Abbreviations

BC	breast cancer
*RASSF1A*	RAS-association domain family member 1A gene
*ATM*	ataxia-telangiectasia mutated gene
450K	Infinium HumanMethylation450 BeadChip
IQR	interquartile range
DCIS	ductal carcinoma in situ
IDC	invasive ductal carcinoma
cfDNA	cell free DNA
CTCs	circulating tumor cells
