# Supplementary material for: Evaluation of Promoter Methylation of RASSF1A and ATM in Peripheral Blood of Breast Cancer Patients and Healthy Control Individuals
Source: Int J Mol Sci. 2018 Mar 19;19(3):900. doi: 10.3390/ijms19030900 (PMC5877761; doi:10.3390/ijms19030900)
Supplement: Supplementary file 1 [file ijms-19-00900-s001.pdf]

Supplementary Tables

Table S1. Gene specific methylation in blood DNA in breast cancer cases and controls investigated in more than one study

Gene	Author, year	Sample	Assay	Case no./ control no.	Position	Case age/ control age (y)[a]	Meth (case)	Meth(control)	*P* values
BRCA1	Cho YH, 2015[1]	white blood cell	MethyLight (%)	1021/1036	promoter	na/na	12	10	*>0.05*
	Gupta S, 2014[2]	whole blood	MS-HRM (%)	66/36	promoter	48.8/56.1	22.7	5.6	*0.03*
	Bosviel R, 2012[3]	whole blood	QMSP (%)	902/990	promoter	47.1/45.9	47.1(46.1-48.1)	45.9(45.0-46.8)	*0.08*
	Wong EM, 2011[4]	whole blood	MS-HRM (%)	255/169	promoter	<40/<40	10.9	3.6	*0.004*
	Iwamoto T, 2011[5]	whole blood	MSP (%)	200/200	promoter	50/50	21.5	13.5	*0.045*
	Snell C, 2008[6]	peripheral blood leukocyte	MethyLight (%)	7/7	promoter	35-51/35-51	42.9	14.3	*<0.05*
	Cho YH, 2010[7]	white blood cell	MethyLight (%)	40/40	promoter	50.8/48.3	8	5	*>0.05*
	Radpour R, 2011[8]	plasma	EpiTyper assay (mean)	36/30	promoter	67/na	0.58[b]	0.30[b]	*<0.0001*
	Liu LM, 2015[9]	serum	Bisulfite sequencing PCR and MS-HRM (%)	36/30	promoter	na/na	10	1.7	*<0.05*
RASSF1A	Cho YH, 2010[7]	white blood cell	MethyLight (%)	40/40	promoter	50.8/48.3	8	3	*>0.05*
	Zmetakova I, 2013[10]	whole blood	pyrosequencing (mean ± SD)	34/50	promoter	41-90/20-78	1.00 ± 0.00	1.04 ± 0.28	*0.475*
	Kloten V, 2013[11]	serum	MS-PCR (%)	136/135	promoter	33-86/33-86	47.1	25.9	*0.0035*
	Zmetakova I, 2013[10]	plasma	pyrosequencing (mean ± SD)	34/50	promoter	41-90/20-78	2.85 ± 3.13	4.02 ± 6.62	*0.404*
	Ahmed I A, 2010[12]	serum	MSP (%)	26/12	promoter	35-73/35-73	69	<10	-
	Brooks JD, 2010[d][13]	serum	QMSP (%)	50/99	promoter	52/51.8	22	17.2	*>0.05*
	Kim JH, 2010[14]	serum	QMSP (%)	119/125	promoter	51/51	32.8	4.8	*0.004*
	Yazici H, 2009 [d][15]	plasma	MSP (%)	61/39	promoter	na/na	18	5	-
	Hoque M, 2006[16]	plasma	QMSP (%)	47/38	promoter	44.9/37.3	32	5	*0.002*
	Van der Auwera I, 2009[17]	serum	QMSP (%)	79/19	promoter	62/39	35	0	*0.002*
	Papadopoulou E, 2006[18]	plasma	MethyLight (%)	50/14	promoter	na/na	26	0	*<0.05*
	Dulaimi E, 2004[19]	serum	MSP (%)	34/20	promoter	57.4/57.4	56	0	*<0.05* [c]
APC	Zmetakova I, 2013[10]	whole blood	pyrosequencing (mean ± SD)	34/50	promoter	41-90/20-78	1.68 ± 1.04	1.28 ± 0.57	*0.082*
	Cho YH, 2010[7]	white blood cell	MethyLight (%)	40/40	promoter	50.8/48.3	0	0	*>0.05*
	Swellam M, 2015[20]	serum	MS-PCR (%)	121/66	promoter	43/40	93.4	0	*<0.0001*
	Zmetakova I, 2013[10]	plasma	pyrosequencing (mean ± SD)	34/50	promoter	41-90/20-78	4.41 ± 7.81	2.53 ± 1.56	*0.060*
	Radpour R, 2011[8]	plasma	EpiTyper assay (mean)	36/30	promoter	67/na	0.39[b]	0.19[b]	*<0.0001*
	Brooks JD, 2010[d][13]	serum	QMSP (%)	49/96	promoter	52/51.8	2	4.2	*>0.05*
	Hoque M, 2006[16]	plasma	QMSP (%)	47/38	promoter	44.9/37.3	17	0	*0.008*
	Van der Auwera I, 2009[17]	serum	QMSP (%)	79/19	promoter	62/39	29	5	*0.03*
	Dulaimi E, 2004[19]	serum	MSP (%)	34/20	promoter	57.4/57.4	29	0	*<0.05* [c]

Table S1. Continued

Gene	Author, year	Sample	Assay	Case no./ control no.	Position	Case age/ control age (y)[a]	Meth (case)	Meth(control)	*P* values
ATM	Brennan K, 2012[21]	white blood cell	pyrosequencing ATM (mvp2a)		intragenic region				
			BGS cohort (mean and IQR)	249/248		54/54	76.8 (70.9–82.7)	76.4 (70.2–80.2)	*0.02*
			EPIC cohort (mean and IQR)	235/283		52/52	75.7 (70.0–80.8)	76.1 (70.5–80.6)	*0.40*
			KConFab cohort (mean and IQR)	156/210		50/60	81.8 (75.8–86.5)	76.9 (71.6–81.5)	*4.87E-06*
			Pyrosequencing ATM (mvp2b)		intragenic region				
			BGS cohort (mean and IQR)	248/234		54/54	91.4 (85.6–95.0)	91.0 (87.0–94.8)	*0.61*
			EPIC cohort (mean and IQR)	240/287		52/52	92.3 (88.3–95.7)	92.2 (87.3–95.2)	*0.36*
			KConFab cohort (mean and IQR)	162/208		50/60	92.3 (82.4–96.5)	92.6 (87.2–96.3)	*0.24*
	Flanagan JM, 2009[22]	white blood cell	pyrosequencing (mean ± SD)	190/190	Gene-body	62.8/62.8	91.4 (72.8-98.4)	89.8 (53.0-98.0)	*0.002*
ESR1	Zmetakova I, 2013[10]	whole blood	pyrosequencing (mean ± SD)	34/50	promoter	41-90/20-78	4.09 ± 1.44	3.22 ± 0.86	*0.026*
	Widschwendter M, 2008[23]	whole blood	MethyLight (%)	320/676	promoter	50-74/50-74	12.2	13.5	*0.645*
	Zmetakova I, 2013[10]	plasma	pyrosequencing (mean ± SD)	34/50	promoter	41-90/20-78	4.18 ± 4.07	5.24 ± 4.33	*0.338*
	Zurita M, 2010[24]	serum	QMSP (%)	77/34	promoter	na/na	0.005[b]	0.085[b]	*>0.05*
	Van der Auwera I, 2009[17]	serum	QMSP (%)	79/19	promoter	62/39	20	10.5	*0.33*
	Martinez-Galan J, 2008[25]	serum	MSP (%)	106/74	promoter	58/42	0.11[b]	0.02[b]	*0.011*
RARB	Cho YH, 2015[1]	white blood cell	MethyLight (%)	1021/1036	promoter	na/na	33	39	*>0.05*
	Cho YH, 2010[7]	white blood cell	MethyLight (%)	40/40	promoter	50.8/48.3	10	10	*>0.05*
	Swellam M, 2015[20]	serum	MS-PCR (%)	121/66	promoter	43/40	95.9	0	*<0.0001*
	Brooks JD, 2010[d][13]	serum	QMSP (%)	45/88	promoter	52/51.8	6.7	1.1	*>0.05*
	Kim JH, 2010[14]	serum	QMSP (%)	119/125	promoter	51/51	86.6	6.4	*<0.001*
	Hoque M, 2006[16]	plasma	QMSP (%)	47/38	promoter	44.9/37.3	26	8	*0.03*
TIMP3	Zmetakova I, 2013[10]	whole blood	pyrosequencing (mean ± SD)	34/50	promoter	41-90/20-78	3.65 ± 2.55	2.50 ± 0.81	*0.036*
	Widschwendter M, 2008[23]	whole blood	MethyLight (%)	320/676	promoter	50-74/50-74	12.5	14.2	*0.511*
	Zmetakova I, 2013[10]	plasma	pyrosequencing (mean ± SD)	34/50	promoter	41-90/20-78	3.97 ± 8.43	3.92 ± 4.54	*0.338*
	Radpour R, 2011[8]	plasma	EpiTyper assay (mean)	36/30	promoter	67/na	0.60[b]	0.50[b]	*<0.0001*
CDH1	Cho YH, 2015[1]	white blood cell	MethyLight (%)	1021/1036	promoter	na/na	58	66	*>0,05*
	Zmetakova I, 2013[10]	whole blood	pyrosequencing (mean ± SD)	34/50	promoter	41-90/20-78	9.64 ± 2.10	9.02 ± 1.60	*0.698*
	Cho YH, 2010[7]	white blood cell	MethyLight (%)	40/40	promoter	50.8/48.3	8	8	*>0,05*

Table S1. Continued

Gene	Author, year	Sample	Assay	Case no./ control no.	Position	Case age/ control age (y)[a]	Meth (case)	Meth(control)	*P* values
GSTP1	Radpour R, 2011[8]	plasma	EpiTyper assay (mean)	36/30	promoter	67/na	0.52[b]	0.39[b]	*0.003*
	Brooks JD, 2010[d][13]	serum	QMSP (%)	50/99	promoter	52/51.8	4	7.1	*>0.05*
	Hoque M, 2006[16]	plasma	QMSP (%)	47/38	promoter	44.9/37.3	26	0	*0.0008*
DAPK	Ahmed I A, 2010[12]	serum	MSP (%)	26/12	promoter	35-73/35-73	88	<10	*<0.05*
	Dulaimi E, 2004[19]	serum	MSP (%)	34/20	promoter	57.4/57.4	35	0	*<0.05*[c]
IGF2	Harrison K, 2015[26]	whole blood	pyrosequencing (mean ± SD)	189/363	DMR2	56/56	48.94 ± 5.61	48.15 ± 5.77	*0.123*
	Ito Y, 2008[27]	whole blood	pyrosequencing (% of loss of methylation)		DMR0 region				
			EPIC-Norfolk cohort	228/460		60.5/60.3	6.6	6.3	*0.91*
			ABC cohort	338/84		52.6/43.2	5.6	7.1	*0.65*
SYK	Zmetakova I, 2013[10]	whole blood	pyrosequencing (mean ± SD)	34/50	promoter	41-90/20-78	1.15 ± 0.44	1.06 ± 0.24	*0.638*
	Widschwendter M, 2008[23]	whole blood	MethyLight (%)	320/676	promoter	50-74/50-74	2.2	2.4	*0.889*

Abbreviations: MSP: methylation-specific PCR; QMSP: quantitative methylation-specific PCR; MS-HRM: methylation-sensitive high-resolution melting; na: not available.

[a] Age indicates mean age or range.

[b] Data was extracted from scatter plots or boxplots in the article.

[c] *P* values were calculated by Fisher's exact test.

[d] Nested case–control study; the others are case–control study.

This table was adapted from Tang, *et al.* 2016[28].

Table S2. *RASSF1A* methylation in sporadic BC patients with different clinical characteristics

Clinical characteristics (N)	Group (N)	Median of Age	Median of methylation levels														
			CpG_1	CpG_8	CpG_9	CpG_11,12	CpG_13	CpG_14,15	CpG_16	CpG_19	CpG_20	CpG_21,22	CpG_23	CpG_24	CpG_25	CpG_26	MEAN
**TNM stage (211)**	stage 0 (1)	46	0.00	0.00	0.00	0.12	0.04	0.12	0.02	0.00	0.00	0.07	0.00	0.04	0.00	0.05	0.033
	stage I(69)	48	0.00	0.00	0.01	0.125	0.04	0.125	0.02	0.00	0.00	0.05	0.00	0.03	0.01	0.04	0.037
	stage II (72)	48.5	0.00	0.00	0.01	0.13	0.02	0.13	0.02	0.00	0.00	0.05	0.00	0.03	0.01	0.04	0.034
	stage III (15)	50	0.00	0.00	0.01	0.15	0.02	0.15	0.02	0.00	0.00	0.04	0.00	0.03	0.01	0.04	0.034
	stage IV (4)	48.5	0.00	0.045	0.00	0.13	0.00	0.13	0.02	0.045	0.045	0.045	0.00	0.04	0.00	0.025	0.043
	neoadj.* (50)	48.5	0.00	0.00	0.01	0.14	0.06	0.14	0.02	0.00	0.00	0.05	0.00	0.04	0.01	0.04	0.039
	P value (Kruskal-Wallis Test)	*0.684*	*0.985*	*0.676*	*0.762*	*0.785*	*0.602*	*0.785*	*0.791*	*0.676*	*0.676*	*0.464*	*0.995*	*0.925*	*0.762*	*0.782*	*0.558*
**Type of BC (209)**	Ductal (179)	49	0.00	0.00	0.01	0.13	0.04	0.13	0.02	0.00	0.00	0.05	0.00	0.03	0.01	0.04	0.037
	Lobular (13)	48	0.00	0.00	0.00	0.11	0.04	0.11	0.02	0.00	0.00	0.04	0.00	0.03	0.00	0.04	0.028
	Ductal-Lobular (3)	46	0.00	0.00	0.01	0.1	0.05	0.1	0.03	0.00	0.00	0.04	0.00	0.04	0.01	0.04	0.021
	DCIS (4)	46	0.00	0.00	0.005	0.12	0.045	0.12	0.02	0.00	0.00	0.035	0.00	0.04	0.005	0.04	0.032
	Others(10)	49.5	0.00	0.00	0.005	0.14	0.09	0.14	0.025	0.00	0.00	0.04	0.00	0.04	0.005	0.04	0.039
	P value (Kruskal-Wallis Test)	*0.776*	*0.887*	*0.987*	*0.323*	*0.299*	*0.339*	*0.299*	*0.477*	*0.987*	*0.987*	*0.216*	*0.987*	*0.819*	*0.323*	*0.649*	*0.209*
**ER status (181)**	ER negative (21)	48	0.00	0.00	0.01	0.12	0.045	0.12	0.03	0.00	0.00	0.04	0.00	0.05	0.01	0.04	0.04
	ER positive (160)	48	0.00	0.00	0.01	0.13	0.04	0.13	0.02	0.00	0.00	0.05	0.00	0.03	0.01	0.04	0.036
	P value (Mann-Whitney U)	*0.254*	*0.334*	*0.503*	*0.903*	*0.936*	*0.751*	*0.936*	*0.247*	*0.503*	*0.503*	*0.854*	*0.605*	*0.061*	*0.903*	*0.745*	*0.369*
**PR status (181)**	PR negative (36)	47	0.00	0.00	0.01	0.12	0.06	0.12	0.02	0.00	0.00	0.04	0.00	0.04	0.01	0.04	0.037
	PR positive (145)	49	0.00	0.00	0.01	0.13	0.03	0.13	0.02	0.00	0.00	0.05	0.00	0.03	0.01	0.04	0.036
	P value (Mann-Whitney U)	*0.060*	*0.178*	*0.815*	*0.327*	*0.652*	*0.298*	*0.652*	*0.988*	*0.815*	*0.815*	*0.227*	*0.477*	*0.281*	*0.327*	*0.441*	*0.948*
**HER2 status (181)**	HER2 negative (165)	48	0.00	0.00	0.01	0.13	0.04	0.13	0.02	0.00	0.00	0.05	0.00	0.03	0.01	0.04	0.036
	HER2 positive (16)	46	0.00	0.00	0.01	0.13	0.05	0.13	0.02	0.00	0.00	0.035	0.00	0.04	0.01	0.035	0.036
	P value (Mann-Whitney U)	*0.292*	*0.410*	*0.324*	*0.932*	*0.614*	*0.901*	*0.614*	*0.549*	*0.324*	*0.324*	*0.133*	*0.657*	*0.977*	*0.923*	*0.533*	*0.526*

* patients underwent neoadjuvant chemotherapy.

Significant *P* values are in bold, α= 0.05.

Table S3. *ATM* methylation in sporadic BC patients with different clinical characteristics

**Clinical characteristics (N)**	**Group (N)**	**Median of Age**	**Median of methylation levels**										
			**CpG_1**	**CpG_2,3,4,5**	**CpG_6**	**CpG_7,8**	**CpG_10,11**	**CpG_12**	**CpG_13,14**	**CpG_17**	**CpG_18,19**	**CpG_20,21**	**CpG_26**
**TNM stage (211)**	stage 0 (1)	46	0.1		0.00	0.03	0.11	0.00	0.08	0.15	0.16	0.02	0.1
	stage I(69)	48	0.01	0.07	0.00	0.05	0.11	0.00	0.03	0.00	0.135	0.02	0.06
	stage II (72)	48.5	0.02	0.06	0.00	0.05	0.12	0.00	0.04	0.00	0.14	0.02	0.07
	stage III (15)	50	0.02	0.09	0.00	0.03	0.11	0.00	0.04	0.02	0.12	0.03	0.08
	stage IV (4)	48.5	0.03	0.07	0.00	0.05	0.11	0.00	0.025	0.015	0.13	0.025	0.055
	neoadj.* (50)	48.5	0.02	0.07	0.00	0.05	0.115	0.00	0.02	0.00	0.13	0.02	0.06
	P value (Kruskal-Wallis Test)	*0.684*	*0.207*	*0.829*	*0.330*	*0.470*	*0.413*	*0.980*	*0.106*	*0.398*	*0.724*	*0.923*	*0.818*
**Type of BC (209)**	Ductal (179)	49	0.02	0.07	0.00	0.05	0.11	0.00	0.03	0.00	0.14	0.02	0.06
	Lobular (13)	48	0.01	0.06	0.00	0.05	0.11	0.00	0.03	0.00	0.13	0.02	0.08
	Ductal-Lobular (3)	46	0.085	0.09	0.00	0.05	0.12	0.00	0.05	0.00	0.12	0.02	0.08
	DCIS (4)	46	0.06	0.09	0.00	0.04	0.11	0.00	0.035	0.06	0.135	0.025	0.1
	Others(10)	49.5	0.015	0.11	0.00	0.05	0.11	0.00	0.02	0.02	0.115	0.03	0.07
	P value (Kruskal-Wallis Test)	*0.776*	*0.385*	*0.152*	*0.971*	*0.906*	*0.433*	*0.638*	*0.086*	*0.081*	*0.313*	*0.736*	*0.270*
**ER status (181)**	ER negative (21)	48	0.025	0.065	0.00	0.05	0.115	0.00	0.04	0.02	0.14	0.03	0.07
	ER positive (160)	48	0.02	0.07	0.00	0.05	0.11	0.00	0.03	0.00	0.13	0.02	0.065
	P value (Mann-Whitney U)	*0.254*	*0.161*	*0.699*	*0.534*	*0.764*	*0.503*	*0.534*	*0.127*	*0.059*	*0.767*	*0.517*	*0.118*
**PR status (181)**	PR negative (36)	47	0.015	0.07	0.00	0.05	0.11	0.00	0.03	0.01	0.13	0.03	0.07
	PR positive (145)	49	0.02	0.07	0.00	0.05	0.11	0.00	0.03	0.00	0.135	0.02	0.06
	P value (Mann-Whitney U)	*0.06*	*0.988*	*0.706*	*0.387*	*0.896*	*0.778*	***0.042***	*0.903*	*0.079*	*0.590*	*0.274*	*0.080*
**HER2 status (181)**	HER2 negative (165)	48	0.02	0.07	0.00	0.05	0.11	0.00	0.03	0.00	0.14	0.02	0.07
	HER2 positive (16)	46	0.01	0.07	0.00	0.05	0.12	0.00	0.03	0.00	0.13	0.02	0.06
	P value (Mann-Whitney U)	*0.292*	*0.660*	*0.625*	*0.583*	*0.302*	*0.724*	*0.143*	*0.963*	*0.915*	*0.596*	*0.829*	*0.681*

* patients underwent neoadjuvant chemotherapy.

Significant *P* values are in bold, α= 0.05.

Table S3. Continued

Clinical characteristics (N)	Group (N)	Median of Age	Median of methylation levels											
			CpG_27	CpG_28	CpG_29	CpG_32	CpG_33	CpG_34	CpG_35	CpG_36	CpG_37	CpG_38	CpG_39	MEAN
**TNM stage (211)**	stage 0 (1)	46	0.1	0.04	0.05	0.00	0.00	0.03	0.15	0.00	0.03		0.00	0.058
	stage Ⅰ(69)	48	0.11	0.02	0.03	0.00	0.00	0.13	0.00	0.00	0.13	0.00	0.02	0.046
	stage Ⅱ (72)	48.5	0.11	0.02	0.03	0.00	0.00	0.13	0.00	0.00	0.13	0.00	0.02	0.050
	stage Ⅲ (15)	50	0.11	0.03	0.03	0.00	0.00	0.12	0.02	0.00	0.12	0.00	0.02	0.050
	stage Ⅳ (4)	48.5	0.115	0.03	0.025	0.00	0.00	0.17	0.015	0.00	0.17	0.005	0.02	0.049
	neoadj.* (50)	48.5	0.11	0.02	0.03	0.00	0.00	0.13	0.00	0.00	0.13	0.00	0.02	0.045
	P value (Kruskal-Wallis Test)	*0.684*	*0.959*	*0.586*	*0.572*	*0.953*	*0.907*	*0.608*	*0.398*	*0.953*	*0.608*	*0.310*	*0.585*	*0.429*
**Type of BC (209)**	Ductal (179)	49	0.11	0.02	0.03	0.00	0.00	0.13	0.00	0.00	0.13	0.00	0.02	0.047
	Lobular (13)	48	0.11	0.02	0.03	0.00	0.00	0.11	0.00	0.00	0.11	0.00	0.02	0.040
	Ductal-Lobular (3)	46	0.12	0.03	0.04	0.00	0.00	0.15	0.00	0.00	0.15	0.00	0.01	0.053
	DCIS (4)	46	0.105	0.025	0.025	0.00	0.00	0.1	0.06	0.00	0.1	0.01	0.04	0.054
	Others(10)	49.5	0.1	0.02	0.03	0.00	0.00	0.12	0.02	0.00	0.12	0.00	0.015	0.047
	P value (Kruskal-Wallis Test)	*0.776*	*0.728*	*0.720*	*0.259*	*0.871*	*0.771*	*0.220*	*0.081*	*0.871*	*0.220*	*0.742*	*0.389*	*0.152*
**ER status (181)**	ER negative (21)	48	0.11	0.03	0.03	0.00	0.00	0.135	0.02	0.00	0.135	0.00	0.03	0.050
	ER positive (160)	48	0.11	0.02	0.03	0.00	0.00	0.13	0.00	0.00	0.13	0.00	0.02	0.047
	P value (Mann-Whitney U)	*0.254*	*0.667*	***0.025***	*0.127*	*0.556*	*0.126*	*0.851*	*0.059*	*0.556*	*0.851*	*0.187*	*0.176*	*0.119*
**PR status (181)**	PR negative (36)	47	0.11	0.03	0.03	0.00	0.00	0.13	0.01	0.00	0.13	0.00	0.03	0.048
	PR positive (145)	49	0.11	0.02	0.03	0.00	0.00	0.13	0.00	0.00	0.13	0.00	0.02	0.046
	P value (Mann-Whitney U)	*0.06*	*0.236*	*0.115*	*0.089*	*0.253*	*0.516*	*0.833*	*0.079*	*0.253*	*0.833*	*0.106*	*0.119*	*0.280*
**HER2 status (181)**	HER2 negative (165)	48	0.11	0.02	0.03	0.00	0.00	0.13	0.00	0.00	0.13	0.00	0.02	0.047
	HER2 positive (16)	46	0.11	0.02	0.03	0.00	0.00	0.10	0.00	0.00	0.10	0.00	0.03	0.043
	P value (Mann-Whitney U)	*0.292*	*0.485*	*0.271*	*0.319*	*0.476*	*0.517*	***0.032***	*0.915*	*0.476*	***0.032***	*0.705*	*0.534*	*0.153*

* patients underwent neoadjuvant chemotherapy.

Significant *P* values are in bold, α= 0.05.

Table S4. Comparison of specific genes data in literature with 450K results

Gene	Literature†					450K results						
	Author, year	Cases No./ Controls No.	Meth (BC cases)	Meth (controls)	P value	CpG	Cases No./ Controls No.	Position	BC Cases Mean±SD	Controls Mean±SD	P^a value	P^b value
BRCA1	Cho YH, 2015[1]	1021/1036	12%	10%	*>0.05*	cg01879757	48/48	3'UTR;Body	0.729±0.068	0.760±0.051	*0.66*	*0.23*
	Gupta S, 2014[2]	66/36	22.7%	5.6%	*0.03*	cg02286533	48/48	TSS1500;Body	0.671±0.092	0.675±0.093	*0.80*	*0.99*
	Bosviel R, 2012[3]	902/990	47.1(46.1-48.1)%	45.9(45.0-46.8)%	*0.08*	cg04110421	48/48	TSS200;5'UTR;1stExon;TSS1500	0.033±0.048	0.025±0.005	*0.81*	*0.64*
	Wong EM, 2011[4]	255/169	10.9%	3.6%	*0.004*	cg04582861	48/48	5'UTR;Body;TSS1500	0.167±0.043	0.182±0.029	*0.23*	*0.96*
	Iwamoto T, 2011[5]	200/200	21.5%	13.5%	*0.045*	cg04658354	48/48	TSS200;5'UTR;1stExon;TSS1500	0.030±0.041	0.025±0.004	*0.91*	*0.77*
	Snell C, 2008[6]	7/7	42.9%	14.3%	*<0.05*	cg05815247	48/48	TSS1500;Body	0.812±0.050	0.822±0.034	*0.64*	*0.83*
	Cho YH, 2010[7]	40/40	8%	5%	*>0.05*	cg06001716	48/48	TSS1500;Body	0.677±0.101	0.672±0.095	*0.92*	*0.91*
	Radpour R, 2011[8]	36/30	0.58	0.30	*<0.0001*	cg06973652	48/48	TSS1500;Body	0.897±0.046	0.908±0.027	*0.64*	*0.71*
	Liu LM, 2015[9]	36/30	10%	1.7%	*<0.05*	cg07054526	48/48	Body;5'UTR	0.908±0.013	0.911±0.007	*0.94*	*0.72*
						cg08386886	48/48	5'UTR;Body;TSS1500	0.089±0.027	0.087±0.018	*0.98*	*0.76*
						cg08993267	48/48	5'UTR;1stExon;Body;TSS1500	0.043±0.031	0.038±0.006	*0.78*	*0.68*
						cg09441966	48/48	TSS200;5'UTR;1stExon;TSS1500	0.026±0.036	0.021±0.003	*0.87*	*0.76*
						cg09831010	48/48	TSS1500;Body	0.043±0.050	0.035±0.009	*0.79*	*0.49*
						cg10609677	48/48	TSS1500;Body	0.582±0.031	0.590±0.028	*0.49*	*0.79*
						cg10893007	48/48	Body;TSS1500;TSS200	0.028±0.031	0.022±0.003	*0.55*	*0.51*
						cg11126247	48/48	TSS1500;Body	0.027±0.027	0.022±0.004	*0.46*	*0.57*
						cg11529738	48/48	TSS1500;Body	0.718±0.055	0.728±0.053	*0.57*	*0.93*
						cg11964474	48/48	TSS1500;Body	0.793±0.032	0.789±0.027	*0.82*	*0.87*
						cg12182452	48/48	TSS1500;Body	0.038±0.046	0.031±0.005	*0.71*	*0.61*
						cg12984107	48/48	5'UTR;Body;TSS1500	0.070±0.048	0.063±0.015	*0.83*	*0.57*
						cg13782816	48/48	Body;5'UTR;1stExon	0.831±0.038	0.856±0.021	*0.43*	***0.043***
						cg14048487	48/48	Body;5'UTR	0.822±0.024	0.826±0.024	*0.63*	*0.70*
						cg14687474	48/48	TSS1500;Body	0.827±0.063	0.837±0.052	*0.64*	*0.89*
						cg14947218	48/48	TSS1500;Body	0.679±0.087	0.687±0.088	*0.80*	*1.00*
						cg15065591	48/48	TSS1500;Body	0.926±0.057	0.936±0.039	*0.63*	*0.93*
						cg15419295	48/48	5'UTR;TSS200;1stExon;TSS1500	0.035±0.029	0.029±0.002	*0.84*	*0.54*

Table S4. Continued

	CpG	Cases No./ Controls No.	Position	BC Cases Mean±SD	Controls Mean±SD	P^a value	P^b value
BRCA1	cg16006004	48/48	TSS1500;Body	0.734±0.108	0.754±0.091	*0.56*	*0.95*
	cg16029534	48/48	Body;3'UTR	0.934±0.008	0.936±0.009	*0.71*	*0.51*
	cg16630982	48/48	5'UTR;TSS200;1stExon;TSS1500	0.030±0.024	0.025±0.003	*0.70*	*0.44*
	cg16919093	48/48	Body	0.884±0.024	0.894±0.015	*0.62*	*0.26*
	cg16963062	48/48	5'UTR;TSS200;1stExon;TSS1500	0.034±0.029	0.029±0.004	*0.99*	*0.53*
	cg17301289	48/48	TSS200;5'UTR;1stExon;TSS1500	0.038±0.039	0.031±0.005	*0.72*	*0.61*
	cg18372208	48/48	TSS1500;Body	0.679±0.094	0.683±0.087	*0.85*	*0.98*
	cg18830083	48/48	Body	0.894±0.013	0.896±0.010	*0.52*	*0.83*
	cg19088651	48/48	5'UTR;Body;TSS1500	0.052±0.030	0.043±0.010	*0.73*	*0.20*
	cg19442659	48/48	TSS1500;Body	0.502±0.048	0.520±0.043	*0.27*	*0.94*
	cg19454999	48/48	TSS1500;Body	0.801±0.075	0.811±0.054	*0.69*	*0.95*
	cg19531713	48/48	5'UTR;Body;TSS1500	0.047±0.045	0.040±0.010	*0.76*	*0.76*
	cg20185525	48/48	TSS1500;Body	0.608±0.022	0.613±0.027	*0.06*	*0.62*
	cg20187250	48/48	5'UTR;TSS200;1stExon;TSS1500	0.020±0.018	0.017±0.002	*0.97*	*0.49*
	cg20760063	48/48	TSS200;TSS1500	0.041±0.043	0.035±0.007	*0.78*	*0.83*
	cg21253966	48/48	TSS200;5'UTR;1stExon;TSS1500	0.031±0.041	0.025±0.003	*0.96*	*0.71*
	cg24806953	48/48	5'UTR;TSS200;1stExon;TSS1500	0.026±0.018	0.023±0.002	*0.92*	*0.79*
	cg24900425	48/48	TSS1500;Body	0.847±0.088	0.856±0.077	*0.64*	*0.96*
	cg25067162	48/48	TSS1500;Body	0.423±0.135	0.404±0.085	*0.99*	*0.80*
	cg25288140	48/48	TSS1500;Body	0.836±0.119	0.857±0.107	*0.47*	*0.88*
	cg25738236	48/48	TSS1500;Body	0.833±0.030	0.830±0.028	*0.87*	*0.79*
	cg26276233	48/48	TSS1500;Body	0.723±0.090	0.735±0.082	*0.63*	*0.99*
	cg26370022	48/48	TSS1500;Body	0.634±0.048	0.637±0.049	*0.75*	*0.96*
	cg26879546	48/48	TSS1500;Body	0.967±0.006	0.968±0.005	*0.28*	*0.86*
	cg26891576	48/48	TSS200;TSS1500	0.055±0.024	0.050±0.009	*0.98*	*0.66*
	cg27383744	48/48	Body	0.835±0.022	0.842±0.023	*0.56*	*0.48*
	cg27581762	48/48	TSS1500;Body	0.756±0.039	0.757±0.036	*0.84*	*0.97*

Table S4. Continued

	Author, year	Cases No./ Controls No.	Meth (BC cases)	Meth (controls)	*P* value	CpG	Cases No./ Controls No.	Position	BC Cases Mean±SD	Controls Mean±SD	P^{a} value	P^{b} value
APC	Zmetakova I, 2013*[10]	34/50	1.68 ± 1.04	1.28 ± 0.57	*0.082*	cg00190738	48/48	TSS1500	0.903±0.010	0.906±0.008	*0.88*	*0.64*
	Cho YH, 2010[7]	40/40	0%	0%	*>0.05*	cg00577935	48/48	TSS1500;5'UTR	0.026±0.004	0.026±0.004	*0.44*	*0.95*
	Swellam M, 2015[20]	121/66	93.4%	0%	*<0.0001*	cg01240931	48/48	5'UTR	0.346±0.036	0.368±0.031	***0.02***	*0.30*
	Zmetakova I, 2013‡[10]	34/50	4.41 ± 7.81	2.53 ± 1.56	*0.06*	cg01528425	48/48	TSS200	0.048±0.007	0.049±0.006	*0.74*	*0.73*
	Radpour R, 2011[8]	36/30	0.39	0.19	*<0.0001*	cg02511809	48/48	TSS200;;5'UTR	0.126±0.019	0.127±0.022	*0.59*	*0.74*
	Brooks JD, 2010[13]	49/96	2%	4.2%	*>0.05*	cg03667968	48/48	TSS200;5'UTR	0.026±0.003	0.025±0.003	*0.93*	*0.28*
	Hoque M, 2006[16]	47/38	17%	0%	*0.008*	cg04011030	48/48	TSS200	0.049±0.008	0.049±0.010	*0.96*	*0.94*
	Van der Auwera I, 2009[17]	79/19	29%	5%	*0.03*	cg04226363	48/48	5'UTR	0.025±0.003	0.025±0.004	*0.92*	*0.82*
	Dulaimi E, 2004[19]	34/20	29%	0%	*<0.05*	cg07003745	48/48	TSS200	0.017±0.002	0.017±0.002	*0.99*	*0.59*
						cg07661636	48/48	TSS1500	0.893±0.019	0.901±0.020	*0.45*	*0.16*
						cg07863043	48/48	Body	0.896±0.013	0.903±0.015	*0.89*	*0.24*
						cg08512345	48/48	5'UTR;1stExon	0.016±0.002	0.016±0.002	*0.72*	*0.86*
						cg08571859	48/48	TSS1500;5'UTR	0.028±0.004	0.027±0.005	*0.92*	*0.56*
						cg08934600	48/48	TSS200	0.030±0.005	0.032±0.005	*0.60*	*0.27*
						cg11057897	48/48	TSS1500	0.373±0.043	0.380±0.044	*0.78*	*0.94*
						cg11479000	48/48	5'UTR	0.881±0.016	0.883±0.014	*0.83*	*0.88*
						cg11613015	48/48	TSS200;5'UTR	0.043±0.006	0.040±0.005	*0.83*	*0.11*
						cg12534150	48/48	1stExon;5'UTR	0.027±0.004	0.026±0.003	*0.92*	*0.69*
						cg14479889	48/48	TSS200;5'UTR	0.028±0.006	0.028±0.005	*0.91*	*0.72*
						cg14511739	48/48	TSS200;5'UTR	0.031±0.004	0.028±0.003	*0.40*	***0.04***
						cg15020645	48/48	5'UTR	0.029±0.007	0.033±0.007	*0.30*	*0.62*
						cg16106903	48/48	Body	0.911±0.012	0.912±0.012	*0.79*	*0.85*
						cg16110711	48/48	TSS1500	0.616±0.040	0.612±0.052	*0.80*	*0.81*
						cg16451027	48/48	TSS1500	0.872±0.021	0.863±0.029	*0.61*	*0.65*
						cg16481008	48/48	TSS200	0.039±0.007	0.039±0.008	*0.91*	*0.83*
						cg16970232	48/48	TSS200;5'UTR	0.033±0.006	0.032±0.005	*0.85*	*0.87*
						cg18315896	48/48	TSS200	0.044±0.006	0.042±0.007	*0.78*	*0.38*
						cg18536802	48/48	TSS200	0.041±0.006	0.040±0.006	*0.78*	*0.86*
						cg19115695	48/48	TSS1500	0.897±0.015	0.904±0.012	*0.40*	*0.11*
						cg20311501	48/48	TSS200;5'UTR	0.089±0.013	0.087±0.013	*0.66*	*0.67*
						cg21634602	48/48	1stExon;5'UTR	0.049±0.011	0.046±0.007	*0.75*	*0.35*
						cg22035501	48/48	TSS200;5'UTR	0.036±0.005	0.034±0.004	*0.93*	*0.11*
						cg23497707	48/48	5'UTR	0.643±0.067	0.681±0.042	*0.06*	*0.74*
						cg23938220	48/48	TSS200;5'UTR	0.016±0.002	0.016±0.002	*0.91*	*0.43*
						cg24332422	48/48	5'UTR	0.052±0.008	0.055±0.008	*0.46*	*0.85*
						cg25645338	48/48	5'UTR	0.904±0.013	0.910±0.009	*0.97*	*0.08*
						cg25922032	48/48	5'UTR;1stExon	0.018±0.003	0.017±0.002	*0.85*	*0.81*
						cg26660754	48/48	TSS200	0.067±0.010	0.067±0.008	*0.66*	*0.84*
						cg27062904	48/48	TSS1500	0.919±0.012	0.925±0.008	*0.47*	*0.07*
						cg27379240	48/48	TSS1500	0.493±0.045	0.497±0.058	*0.95*	*0.99*

Table S4. Continued

	Author, year	Cases No./ Controls No.	Meth (BC cases)	Meth (controls)	*P* value	CpG	Cases No./ Controls No.	Position	BC Cases Mean±SD	Controls Mean±SD	P^a value	P^b value
ESR1	Zmetakova I, 2013*[10]	34/50	4.09 ± 1.44	3.22 ± 0.86	*0.026*	cg00601836	48/48	Body	0.902±0.035	0.892±0.044	*0.91*	*0.76*
	Widschwendter M, 2008[23]	320/676	12.2%	13.5%	*0.645*	cg00655307	48/48	5'UTR;TSS200	0.068±0.017	0.070±0.013	*0.98*	*0.46*
	Zmetakova I, 2013‡[10]	34/50	4.18 ± 4.07	5.24 ± 4.33	*0.338*	cg00920970	48/48	Body;1stExon	0.029±0.004	0.029±0.004	*0.93*	*0.59*
	Zurita M, 2010[24]	77/34	0.005	0.085	*>0.05*	cg01321962	48/48	5'UTR;TSS1500	0.931±0.007	0.932±0.007	*0.77*	*0.94*
	Van der Auwera I, 2009[17]	79/19	20%	10.5%	*0.33*	cg01715172	48/48	TSS1500;5'UTR	0.836±0.027	0.848±0.015	*0.95*	*0.26*
	Martinez-Galan J, 2008[25]	106/74	0.11	0.02	*0.011*	cg01777019	48/48	5'UTR;TSS200	0.036±0.008	0.036±0.007	*0.93*	*0.80*
						cg02285263	48/48	Body	0.040±0.009	0.040±0.008	*0.96*	*0.46*
						cg02404255	48/48	Body	0.924±0.008	0.924±0.009	*0.84*	*0.96*
						cg02720618	48/48	Body	0.031±0.009	0.030±0.008	*0.96*	*0.29*
						cg03037684	48/48	3'UTR	0.550±0.027	0.554±0.025	*0.66*	*0.94*
						cg03732055	48/48	Body	0.918±0.012	0.923±0.008	*0.95*	*0.10*
						cg04063345	48/48	Body	0.791±0.053	0.786±0.083	*0.94*	*0.89*
						cg04211581	48/48	1stExon;5'UTR	0.080±0.025	0.091±0.048	*0.58*	*0.66*
						cg05171584	48/48	TSS1500;5'UTR;1stExon	0.133±0.040	0.146±0.028	*0.30*	*0.70*
						cg06611115	48/48	TSS1500	0.896±0.017	0.895±0.019	*0.92*	*0.93*
						cg06877423	48/48	Body	0.841±0.030	0.853±0.028	*0.42*	*0.51*
						cg07059469	48/48	3'UTR	0.332±0.054	0.354±0.046	*0.38*	*0.80*
						cg07189962	48/48	5'UTR;TSS1500	0.907±0.015	0.912±0.007	*0.99*	*0.41*
						cg07455133	48/48	Body	0.845±0.029	0.824±0.040	*0.08*	*0.83*
						cg07584093	48/48	5'UTR;TSS1500	0.908±0.011	0.911±0.008	*0.79*	*0.37*
						cg07619683	48/48	5'UTR;TSS1500	0.884±0.025	0.895±0.017	*0.51*	*0.21*
						cg07671949	48/48	TSS1500;TSS200;5'UTR	0.118±0.023	0.120±0.021	*0.73*	*0.70*
						cg07746998	48/48	5'UTR;TSS200	0.695±0.048	0.686±0.038	*0.71*	*0.64*
						cg08161546	48/48	TSS1500	0.384±0.113	0.418±0.099	*0.62*	*0.79*
						cg08415493	48/48	5'UTR	0.860±0.026	0.852±0.023	*0.74*	*0.77*
						cg08884395	48/48	TSS1500;5'UTR	0.880±0.020	0.875±0.031	*0.60*	*0.85*
						cg08907436	48/48	5'UTR;TSS1500	0.938±0.008	0.934±0.011	*0.56*	*0.72*
						cg09414638	48/48	Body	0.871±0.029	0.877±0.015	*0.80*	*0.65*
						cg09646983	48/48	5'UTR;TSS1500	0.933±0.007	0.935±0.007	*0.94*	*0.75*
						cg10441070	48/48	5'UTR;TSS1500	0.881±0.025	0.863±0.030	*0.06*	*0.32*
						cg10939667	48/48	Body	0.924±0.010	0.923±0.010	*0.86*	*0.92*
						cg11251858	48/48	5'UTR;1stExon	0.087±0.026	0.089±0.029	*1.00*	*1.00*
						cg11813455	48/48	TSS1500;5'UTR;1stExon	0.065±0.016	0.067±0.014	*0.86*	*0.79*
						cg12209876	48/48	Body	0.941±0.006	0.942±0.006	*0.95*	*0.39*

Table S4. Continued

	CpG	Cases No./ Controls No.	Position	BC Cases Mean±SD	Controls Mean±SD	P^{a} value	P^{b} value
ESR1	cg13612689	48/48	5'UTR;TSS200;1stExon	0.055±0.019	0.056±0.010	*0.96*	*0.99*
	cg15543523	48/48	TSS1500;5'UTR	0.930±0.010	0.934±0.007	*0.94*	*0.24*
	cg15626350	48/48	Body	0.716±0.073	0.703±0.099	*0.89*	*0.71*
	cg15980539	48/48	5'UTR;1stExon	0.022±0.018	0.022±0.011	*0.62*	*0.97*
	cg17264271	48/48	5'UTR;1stExon	0.635±0.045	0.629±0.043	*0.95*	*0.70*
	cg17706972	48/48	5'UTR;TSS1500	0.921±0.011	0.923±0.013	*0.76*	*0.68*
	cg17741339	48/48	5'UTR	0.251±0.038	0.248±0.050	*0.89*	*0.35*
	cg18007957	48/48	1stExon;5'UTR	0.070±0.031	0.076±0.049	*0.64*	*0.92*
	cg18132851	48/48	5'UTR	0.192±0.057	0.200±0.054	*0.67*	*0.46*
	cg18745416	48/48	TSS1500	0.581±0.072	0.606±0.058	*0.61*	*0.82*
	cg19369424	48/48	Body	0.907±0.011	0.910±0.009	*0.97*	*0.53*
	cg19411146	48/48	TSS1500;5'UTR;1stExon	0.041±0.009	0.042±0.012	*0.84*	*0.51*
	cg19449067	48/48	TSS1500	0.668±0.067	0.698±0.056	*0.39*	*0.75*
	cg20253551	48/48	Body;1stExon	0.036±0.010	0.035±0.007	*0.98*	*0.86*
	cg20627916	48/48	TSS1500;TSS200;5'UTR	0.118±0.021	0.119±0.021	*0.88*	*0.48*
	cg20893956	48/48	5'UTR;TSS200	0.698±0.054	0.681±0.057	*0.70*	*0.50*
	cg21157690	48/48	5'UTR;1stExon	0.733±0.042	0.709±0.049	*0.27*	*0.18*
	cg21265702	48/48	Body	0.884±0.018	0.883±0.016	*0.99*	*0.99*
	cg21608605	48/48	TSS1500;TSS200;5'UTR	0.056±0.012	0.056±0.010	*0.87*	*0.51*
	cg21614759	48/48	TSS1500;TSS200;5'UTR	0.025±0.007	0.025±0.005	*0.87*	*0.56*
	cg21950534	48/48	TSS1500;5'UTR;1stExon	0.076±0.019	0.077±0.014	*0.98*	*0.58*
	cg22157087	48/48	5'UTR	0.476±0.115	0.493±0.111	*0.86*	*0.82*
	cg22839866	48/48	TSS1500;5'UTR;1stExon	0.066±0.012	0.067±0.009	*0.86*	*0.71*
	cg23009221	48/48	TSS1500;5'UTR;1stExon	0.105±0.014	0.109±0.011	*0.69*	*0.97*
	cg23164938	48/48	TSS1500;TSS200;5'UTR	0.060±0.012	0.057±0.012	*0.81*	*0.23*
	cg23165623	48/48	TSS1500;TSS200;5'UTR	0.055±0.017	0.057±0.013	*0.80*	*0.55*
	cg23467008	48/48	TSS1500;5'UTR;1stExon	0.019±0.005	0.018±0.004	*0.94*	*0.62*
	cg24764793	48/48	5'UTR;TSS200	0.874±0.039	0.865±0.038	*0.87*	*0.86*
	cg24900983	48/48	TSS1500;5'UTR;1stExon	0.129±0.038	0.139±0.030	*0.58*	*0.69*
	cg25338972	48/48	TSS1500	0.860±0.027	0.875±0.018	*0.31*	*0.18*
	cg25490334	48/48	Body	0.912±0.012	0.911±0.012	*0.83*	*0.76*
	cg25565730	48/48	5'UTR	0.199±0.043	0.223±0.030	***0.02***	*0.38*
	cg26089753	48/48	TSS1500;5'UTR	0.911±0.013	0.915±0.010	*0.82*	*0.46*
	cg27316393	48/48	5'UTR;TSS200	0.055±0.021	0.061±0.024	*0.65*	*0.95*
	ch.6.2949012F	48/48	5'UTR	0.086±0.045	0.065±0.041	*0.18*	*0.09*

Table S4. Continued

	Author, year	Cases No./ Controls No.	Meth (BC cases)	Meth (controls)	*P* value	CpG	Cases No./ Controls No.	Position	BC Cases Mean±SD	Controls Mean±SD	P^a value	P^b value
RARB	Cho YH, 2015[1]	1021/1036	33%	39%	*> 0.05*	cg00371702	48/48	TSS1500	0.274±0.042	0.269±0.042	*0.89*	*0.87*
	Cho YH, 2010[7]	40/40	10%	10%	*> 0.05*	cg00758229	48/48	TSS1500	0.076±0.015	0.080±0.017	*0.59*	*0.61*
	Swellam M, 2015[20]	121/66	95.9%	0%	*< 0.0001*	cg01697477	48/48	Body	0.331±0.081	0.374±0.060	*0.28*	*0.36*
	Brooks JD, 2010[13]	45/88	6.7%	1.1%	*> 0.05*	cg01794805	48/48	5'UTR;Body	0.582±0.092	0.585±0.087	*0.93*	*0.93*
	Kim JH, 2010[14]	119/125	86.6%	6.4%	*< 0.001*	cg02499249	48/48	TSS200	0.055±0.010	0.049±0.012	*0.73*	*0.14*
	Hoque M, 2006[16]	47/38	26%	8%	*0.03*	cg02746691	48/48	Body	0.907±0.010	0.910±0.010	*0.80*	*0.35*
						cg03428864	48/48	5'UTR;1stExon	0.125±0.028	0.134±0.020	*0.30*	*0.92*
						cg03481274	48/48	TSS200	0.121±0.031	0.113±0.032	*0.90*	*0.80*
						cg03531687	48/48	3'UTR	0.793±0.027	0.808±0.021	*0.29*	*0.50*
						cg06705767	48/48	5'UTR;Body	0.781±0.044	0.767±0.036	*0.73*	*0.83*
						cg06720425	48/48	TSS200	0.056±0.007	0.056±0.007	*0.90*	*0.96*
						cg07405178	48/48	Body	0.172±0.039	0.163±0.025	*0.94*	*0.62*
						cg10712623	48/48	TSS1500;TSS200	0.106±0.011	0.107±0.011	*0.99*	*0.82*
						cg11151405	48/48	Body	0.246±0.084	0.272±0.072	*0.30*	*0.84*
						cg12479047	48/48	TSS1500;TSS200	0.019±0.004	0.020±0.003	*0.44*	*0.87*
						cg15011775	48/48	5'UTR;Body	0.687±0.041	0.687±0.047	*0.94*	*0.92*
						cg16927871	48/48	5'UTR;1stExon	0.067±0.012	0.065±0.010	*0.90*	*0.57*
						cg18094781	48/48	TSS1500	0.056±0.022	0.062±0.023	*0.68*	*0.99*
						cg19003815	48/48	5'UTR;1stExon	0.085±0.025	0.088±0.015	*0.81*	*0.90*
						cg20899354	48/48	TSS1500	0.019±0.003	0.020±0.003	*0.49*	*0.92*
						cg20981919	48/48	Body	0.727±0.037	0.732±0.024	*0.98*	*0.71*
						cg21646032	48/48	Body	0.653±0.050	0.661±0.038	*0.86*	*0.94*
						cg21902772	48/48	TSS1500	0.857±0.027	0.843±0.042	*0.41*	*0.36*
						cg22231424	48/48	5'UTR;Body	0.901±0.016	0.898±0.017	*0.42*	*0.85*
						cg23518541	48/48	5'UTR;Body	0.777±0.042	0.763±0.045	*0.67*	*0.95*
						cg24396624	48/48	5'UTR;1stExon	0.059±0.010	0.061±0.011	*0.56*	*0.99*
						cg26124016	48/48	TSS1500	0.020±0.007	0.023±0.010	*0.41*	*0.68*
						cg26786980	48/48	5'UTR;1stExon	0.081±0.012	0.075±0.011	*0.75*	*0.17*
						cg27486427	48/48	5'UTR;1stExon	0.103±0.027	0.111±0.019	*0.42*	*0.83*
						cg27574595	48/48	Body	0.646±0.060	0.642±0.059	*0.87*	*0.62*

Table S4. Continued

	Author, year	Cases No./ Controls No.	Meth (BC cases)	Meth (controls)	*P* value	CpG	Cases No./ Controls No.	Position	BC Cases Mean±SD	Controls Mean±SD	P^{a} value	P^{b} value
TIMP3	Zmetakova I, 2013*[10]	34/50	3.65 ± 2.55	2.50 ± 0.81	*0.036*	cg00936547	48/48	Body;TSS1500	0.937±0.007	0.939±0.006	*0.99*	*0.39*
	Widschwendter M, 2008[23]	320/676	12.5%	14.2%	*0.511*	cg01350190	48/48	Body;TSS1500	0.903±0.013	0.906±0.013	*0.94*	*0.83*
	Zmetakova I, 2013‡[10]	34/50	3.97 ± 8.43	3.92 ± 4.54	*0.338*	cg03356866	48/48	Body;3'UTR	0.880±0.024	0.887±0.019	*0.86*	*0.43*
	Radpour R, 2011[8]	36/30	0.60	0.50	*<0.0001*	cg05260966	48/48	Body;1stExon;5'UTR	0.356±0.030	0.359±0.031	*0.95*	*0.95*
						cg05288803	48/48	Body;1stExon	0.043±0.007	0.043±0.008	*0.98*	*0.76*
						cg05470389	48/48	Body;1stExon;5'UTR	0.026±0.004	0.026±0.005	*0.84*	*0.61*
						cg07641497	48/48	Body;TSS1500	0.802±0.031	0.804±0.028	*0.79*	*0.58*
						cg07972762	48/48	Body;TSS1500	0.759±0.138	0.749±0.129	*0.99*	*0.87*
						cg08613327	48/48	Body;TSS1500	0.909±0.012	0.916±0.009	*0.32*	*0.06*
						cg08687052	48/48	Body;TSS1500	0.929±0.009	0.929±0.006	*0.89*	*0.79*
						cg12498887	48/48	Body;TSS1500	0.872±0.018	0.866±0.030	*0.85*	*0.78*
						cg14456116	48/48	Body;TSS1500	0.905±0.013	0.900±0.015	*0.50*	*0.28*
						cg15004938	48/48	Body;TSS1500	0.879±0.022	0.862±0.026	*0.08*	*0.18*
						cg17571207	48/48	Body;3'UTR	0.523±0.047	0.535±0.044	*0.58*	*0.41*
						cg20500237	48/48	Body;1stExon;5'UTR	0.036±0.005	0.034±0.003	*0.49*	*0.37*
						cg20761450	48/48	Body;TSS1500	0.925±0.007	0.928±0.006	*0.97*	*0.58*
						cg22687380	48/48	Body;TSS200	0.888±0.013	0.891±0.009	*0.93*	*0.54*
						cg23601468	48/48	Body	0.915±0.014	0.912±0.016	*0.81*	*0.82*
						cg23817297	48/48	Body;TSS1500	0.921±0.011	0.920±0.012	*0.95*	*0.98*
						cg24080529	48/48	Body;1stExon;5'UTR	0.523±0.030	0.525±0.027	*0.88*	*0.79*
						cg25245338	48/48	Body;TSS1500	0.925±0.008	0.921±0.012	*0.08*	*0.28*
						cg27221424	48/48	Body;TSS1500	0.881±0.015	0.878±0.054	*0.94*	*0.90*

Table S4. Continued

	Author, year	Cases No./ Controls No.	Meth (BC cases)	Meth (controls)	*P* value	CpG	Cases No./ Controls No.	Position	BC Cases Mean±SD	Controls Mean±SD	P^a value	P^b value
CDH1	Cho YH, 2015[1]	1021/1036	58%	66%	> *0.05*	cg00935351	48/48	TSS1500	0.757±0.027	0.757±0.034	*0.72*	*0.81*
	Zmetakova I, 2013*[10]	34/50	9.64 ± 2.10	9.02 ± 1.60	*0.698*	cg01251360	48/48	Body	0.557±0.055	0.571±0.058	*0.62*	*0.26*
	Cho YH, 2010[7]	40/40	8%	8%	> *0.05*	cg01857829	48/48	Body	0.076±0.022	0.083±0.023	*0.64*	*0.89*
						cg04398983	48/48	TSS200	0.099±0.014	0.103±0.016	*0.53*	*0.89*
						cg05785947	48/48	TSS200	0.035±0.011	0.035±0.009	*0.81*	*0.64*
						cg06875305	48/48	3'UTR	0.817±0.026	0.821±0.024	*0.85*	*0.25*
						cg07762788	48/48	Body	0.921±0.014	0.922±0.012	*0.99*	*0.97*
						cg08051386	48/48	Body	0.922±0.008	0.922±0.008	*0.87*	*0.92*
						cg08616585	48/48	Body	0.643±0.033	0.629±0.035	*0.31*	*0.97*
						cg09220040	48/48	Body	0.924±0.009	0.924±0.008	*0.51*	*0.96*
						cg09406989	48/48	Body	0.613±0.053	0.624±0.055	*0.74*	*0.41*
						cg10313337	48/48	Body	0.364±0.075	0.418±0.060	*0.02*	*0.54*
						cg11255163	48/48	5'UTR;1stExon	0.060±0.009	0.061±0.010	*0.89*	*0.88*
						cg11667754	48/48	TSS1500	0.147±0.034	0.157±0.050	*0.64*	*0.97*
						cg13920367	48/48	TSS200	0.203±0.043	0.212±0.044	*0.81*	*0.89*
						cg16739895	48/48	TSS200	0.020±0.003	0.021±0.003	*0.35*	*0.89*
						cg17655614	48/48	TSS1500	0.312±0.032	0.322±0.048	*0.65*	*0.70*
						cg20716119	48/48	Body	0.136±0.023	0.145±0.018	*0.20*	*0.40*
						cg22832044	48/48	Body	0.077±0.010	0.077±0.011	*0.79*	*0.99*
						cg23989635	48/48	5'UTR;1stExon	0.084±0.011	0.086±0.011	*0.57*	*0.93*
						cg24765079	48/48	Body	0.726±0.050	0.736±0.053	*0.77*	*0.30*
						cg26508465	48/48	Body	0.829±0.028	0.849±0.025	***0.03***	*0.26*

Table S4. Continued

	Author, year	Cases No./ Controls No.	Meth (BC cases)	Meth (controls)	*P* value	CpG	Cases No./ Controls No.	Position	BC Cases Mean±SD	Controls Mean±SD	P^a value	P^b value
GSTP1	Radpour R, 2011[8]	36/30	0.52	0.39	*0.003*	cg02659086	48/48	TSS200	0.017±0.002	0.017±0.002	*0.88*	*0.39*
	Brooks JD, 2010[13]	50/99	4%	7.1%	*> 0.05*	cg04920951	48/48	1stExon;5'UTR	0.012±0.001	0.012±0.001	*0.94*	*0.38*
	Hoque M, 2006[16]	47/38	26%	0%	*0.0008*	cg05244766	48/48	TSS1500	0.882±0.023	0.882±0.019	*0.60*	*0.98*
						cg06841499	48/48	TSS1500	0.310±0.054	0.355±0.057	***0.03***	*0.35*
						cg06928838	48/48	Body	0.041±0.010	0.045±0.010	*0.08*	*0.62*
						cg08925882	48/48	TSS1500	0.825±0.038	0.818±0.039	*0.43*	*0.70*
						cg09038676	48/48	Body	0.031±0.007	0.032±0.009	*0.98*	*0.87*
						cg09657136	48/48	TSS1500	0.464±0.025	0.467±0.026	*0.90*	*0.91*
						cg10552496	48/48	Body	0.843±0.017	0.842±0.014	*0.99*	*0.99*
						cg11566244	48/48	Body	0.117±0.047	0.146±0.050	*0.19*	*0.57*
						cg14567424	48/48	TSS1500	0.494±0.086	0.549±0.084	*0.18*	*0.99*
						cg14921275	48/48	TSS1500	0.614±0.039	0.615±0.039	*0.50*	*0.85*
						cg19114214	48/48	TSS1500	0.251±0.060	0.284±0.068	*0.16*	*0.98*
						cg21734168	48/48	Body	0.936±0.013	0.938±0.009	*0.79*	*0.71*
						cg22224704	48/48	Body	0.295±0.037	0.322±0.032	*0.03*	*0.67*
						cg23725454	48/48	TSS1500	0.269±0.093	0.283±0.077	*0.66*	*0.90*
						cg25135322	48/48	TSS1500	0.236±0.041	0.241±0.043	*0.98*	*0.90*
						cg25866895	48/48	TSS1500	0.537±0.093	0.514±0.085	*0.57*	*0.60*
						cg26250609	48/48	1stExon;5'UTR	0.133±0.023	0.139±0.023	*0.72*	*0.60*

Table S4. Continued

	Author, year	Cases No./ Controls No.	Meth (BC cases)	Meth (controls)	*P* value	CpG	Cases No./ Controls No.	Position	BC Cases Mean±SD	Controls Mean±SD	P^a value	P^b value
DAPK	Ahmed I A, 2010[12]	26/12	88%	<10%	*<0.05*	***DAPK1***						
	Dulaimi E, 2004[19]	34/20	35%	0%	*<0.05*	cg01463032	48/48	Body	0.915±0.011	0.912±0.015	*0.82*	*0.90*
						cg02100497	48/48	Body	0.857±0.022	0.847±0.022	*0.49*	*0.60*
						cg05475556	48/48	Body	0.863±0.018	0.860±0.017	*0.46*	*0.99*
						cg08719486	48/48	TSS1500	0.427±0.062	0.442±0.056	*0.73*	*0.98*
						cg08797471	48/48	5'UTR	0.051±0.016	0.050±0.011	*0.90*	*0.82*
						cg13527872	48/48	Body	0.165±0.032	0.158±0.026	*0.84*	*0.40*
						cg13752933	48/48	Body	0.103±0.030	0.119±0.032	*0.43*	*1.00*
						cg13765778	48/48	Body	0.812±0.034	0.813±0.031	*1.00*	*0.76*
						cg13778339	48/48	Body	0.870±0.015	0.872±0.013	*0.59*	*0.41*
						cg13805297	48/48	Body	0.893±0.016	0.888±0.018	*0.66*	*0.86*
						cg13814950	48/48	TSS1500	0.024±0.004	0.024±0.004	*0.93*	*0.80*
						cg13823120	48/48	TSS1500	0.210±0.037	0.230±0.033	*0.14*	*0.75*
						cg13932603	48/48	TSS1500	0.020±0.002	0.020±0.002	*0.49*	*0.93*
						cg13964439	48/48	Body	0.787±0.019	0.788±0.020	*0.89*	*0.75*
						cg14014720	48/48	Body	0.078±0.019	0.079±0.019	*0.96*	*0.75*
						cg14071249	48/48	Body	0.836±0.037	0.857±0.033	*0.62*	*0.16*
						cg14089032	48/48	Body	0.936±0.008	0.935±0.008	*0.84*	*0.96*
						cg14134019	48/48	Body	0.678±0.025	0.679±0.032	*0.56*	*0.96*
						cg14159523	48/48	Body	0.784±0.029	0.795±0.029	*1.00*	*0.20*
						cg14250336	48/48	Body	0.912±0.009	0.913±0.010	*0.95*	*0.65*
						cg14286732	48/48	Body	0.787±0.032	0.786±0.037	*1.00*	*0.83*
						cg15746719	48/48	5'UTR	0.018±0.004	0.017±0.002	*0.98*	*0.29*
						cg17090012	48/48	Body	0.909±0.010	0.910±0.009	*0.90*	*0.81*
						cg17984409	48/48	Body	0.938±0.010	0.935±0.012	*0.51*	*0.71*
						cg19734228	48/48	5'UTR	0.070±0.012	0.073±0.015	*0.77*	*0.99*
						cg20401521	48/48	TSS200	0.018±0.006	0.020±0.010	*0.69*	*0.98*
						cg21180703	48/48	Body	0.812±0.196	0.846±0.115	*0.65*	*0.91*
						cg22571217	48/48	TSS1500	0.034±0.005	0.035±0.007	*0.80*	*0.93*
						cg24754277	48/48	Body	0.204±0.032	0.217±0.035	*0.47*	*0.97*

Table S4. Continued

	CpG	Cases No./ Controls No.	Position	BC Cases Mean±SD	Controls Mean±SD	P^{a} value	P^{b} value
DAPK	***DAPK3***						
	cg00473257	48/48	Body	0.918±0.010	0.921±0.013	*0.54*	*0.35*
	cg00771170	48/48	TSS1500	0.028±0.002	0.028±0.003	*0.68*	*0.97*
	cg03110167	48/48	Body	0.911±0.011	0.915±0.010	*0.59*	*0.46*
	cg03752885	48/48	1stExon;5'UTR	0.916±0.012	0.920±0.010	*0.80*	*0.44*
	cg04284660	48/48	Body	0.948±0.006	0.949±0.007	*0.97*	*0.92*
	cg05204981	48/48	Body	0.844±0.019	0.844±0.020	*0.88*	*0.98*
	cg06539494	48/48	Body	0.850±0.021	0.846±0.015	*0.75*	*0.56*
	cg06647026	48/48	Body	0.983±0.004	0.983±0.004	*0.79*	*0.90*
	cg06807379	48/48	TSS1500	0.020±0.002	0.021±0.002	*0.11*	*0.46*
	cg07790807	48/48	Body	0.882±0.019	0.884±0.021	*1.00*	*0.76*
	cg08224888	48/48	Body	0.794±0.016	0.799±0.017	*0.54*	*0.71*
	cg08294750	48/48	TSS1500	0.052±0.012	0.052±0.012	*0.77*	*0.97*
	cg13144588	48/48	TSS200	0.302±0.050	0.295±0.059	*0.76*	*0.92*
	cg14748455	48/48	Body	0.760±0.019	0.765±0.014	*0.41*	*0.51*
	cg16256106	48/48	TSS1500	0.047±0.006	0.048±0.007	*0.70*	*0.95*
	cg18226382	48/48	Body	0.878±0.016	0.876±0.014	*0.96*	*0.91*
	cg18459205	48/48	3'UTR	0.913±0.019	0.916±0.019	*0.79*	*0.76*
	cg18707867	48/48	TSS1500	0.028±0.004	0.027±0.003	*0.94*	*0.64*
	cg18748062	48/48	Body	0.920±0.009	0.916±0.011	*0.30*	*0.61*
	cg19109007	48/48	Body	0.936±0.009	0.934±0.012	*0.84*	*0.73*
	cg22304239	48/48	Body	0.893±0.013	0.892±0.013	*0.96*	*0.88*
	cg23837683	48/48	3'UTR	0.927±0.013	0.929±0.013	*0.69*	*0.99*
	cg26585416	48/48	1stExon;5'UTR	0.920±0.009	0.920±0.009	*0.75*	*0.93*
	cg26924890	48/48	TSS1500	0.028±0.005	0.029±0.003	*0.78*	*0.85*
	cg27028514	48/48	Body	0.869±0.058	0.877±0.014	*0.85*	*0.73*

Table S4. Continued

	Author, year	Cases No./ Controls No.	Meth (BC cases)	Meth (controls)	P value	CpG	Cases No./ Controls No.	Position	BC Cases Mean±SD	Controls Mean±SD	P^a value	P^b value
IGF2	Harrison K, 2015[26]	189/363	48.94 ± 5.61	48.15 ± 5.77	*0.123*	cg00221747	48/48	Body;3'UTR	0.986±0.002	0.987±0.002	*0.98*	*0.71*
	Ito Y, 2008[27]					cg00273464	48/48	Body;1stExon;5'UTR	0.769±0.059	0.768±0.048	*0.99*	*0.95*
	EPIC-Norfolk cohort	228/460	6.6	6.3	*0.91*	cg00570518	48/48	Body	0.526±0.044	0.524±0.032	*0.99*	*0.99*
	ABC cohort	338/84	5.6	7.1	*0.65*	cg01351425	48/48	Body;1stExon;5'UTR	0.655±0.035	0.657±0.040	*0.95*	*0.88*
						cg01368777	48/48	Body;5'UTR;TSS200	0.162±0.021	0.163±0.021	*0.96*	*0.98*
						cg01667319	48/48	Body;TSS1500;5'UTR	0.068±0.015	0.070±0.015	*0.97*	*0.96*
						cg01668279	48/48	TSS1500;Body	0.870±0.018	0.869±0.018	*0.94*	*0.90*
						cg01921126	48/48	TSS200;Body	0.682±0.070	0.691±0.065	*0.89*	*0.79*
						cg02045936	48/48	Body;3'UTR	0.886±0.015	0.887±0.014	*0.84*	*0.99*
						cg02166532	48/48	Body;5'UTR;1stExon	0.113±0.011	0.112±0.012	*0.90*	*0.29*
						cg02425416	48/48	Body;5'UTR;TSS1500	0.196±0.045	0.191±0.052	*0.67*	*0.76*
						cg02613624	48/48	Body	0.567±0.040	0.573±0.035	*0.79*	*0.66*
						cg02719427	48/48	Body;3'UTR	0.974±0.002	0.974±0.002	*0.97*	*0.93*
						cg02807948	48/48	Body;3'UTR	0.492±0.048	0.492±0.042	*0.95*	*1.00*
						cg02808220	48/48	TSS1500;Body	0.835±0.017	0.840±0.018	*0.61*	*0.68*
						cg02835822	48/48	Body;TSS1500;5'UTR	0.115±0.019	0.121±0.032	*0.93*	*0.87*
						cg03553386	48/48	TSS200;Body	0.720±0.032	0.709±0.039	*0.51*	*0.60*
						cg03760951	48/48	Body;TSS1500;5'UTR	0.116±0.036	0.118±0.029	*0.94*	*0.84*
						cg04057455	48/48	Body;TSS1500	0.508±0.035	0.483±0.071	*0.13*	*0.70*
						cg04072545	48/48	Body;TSS1500;5'UTR	0.250±0.032	0.252±0.042	*0.76*	*0.84*
						cg04112019	48/48	Body;5'UTR	0.071±0.015	0.072±0.015	*0.61*	*0.88*
						cg05203776	48/48	Body;5'UTR;TSS1500	0.155±0.022	0.151±0.016	*0.76*	*0.63*
						cg05323345	48/48	Body;5'UTR;TSS1500	0.245±0.081	0.220±0.060	*0.46*	*0.66*
						cg05384664	48/48	Body	0.562±0.044	0.563±0.030	*0.98*	*0.90*
						cg05444816	48/48	Body;5'UTR;TSS200	0.079±0.021	0.083±0.016	*0.65*	*0.98*
						cg05452899	48/48	Body;TSS1500;5'UTR	0.086±0.020	0.086±0.019	*0.98*	*0.52*
						cg05777976	48/48	Body;5'UTR;TSS1500	0.056±0.028	0.053±0.020	*0.80*	*0.69*
						cg05859777	48/48	Body;TSS1500;5'UTR	0.072±0.020	0.071±0.019	*0.99*	*0.72*
						cg06029905	48/48	Body;TSS200	0.739±0.022	0.734±0.027	*0.99*	*0.80*
						cg06460568	48/48	Body;5'UTR;1stExon	0.032±0.013	0.032±0.008	*0.73*	*0.98*
						cg06676088	48/48	Body;3'UTR	0.855±0.015	0.846±0.058	*0.76*	*0.95*
						cg07096953	48/48	Body	0.312±0.076	0.295±0.067	*0.91*	*0.79*
						cg07583420	48/48	Body;5'UTR	0.035±0.007	0.035±0.008	*0.94*	*0.88*
						cg08014499	48/48	Body;5'UTR;TSS1500	0.168±0.033	0.183±0.050	*0.56*	*0.77*
						cg08162473	48/48	Body;TSS1500;5'UTR	0.026±0.006	0.027±0.006	*0.71*	*0.91*
						cg08686462	48/48	Body;TSS1500	0.381±0.033	0.374±0.028	*0.69*	*0.98*
						cg08986368	48/48	Body;5'UTR	0.051±0.010	0.053±0.012	*0.77*	*0.84*
						cg09503234	48/48	Body;3'UTR	0.967±0.003	0.967±0.004	*0.92*	*0.94*

Table S4. Continued

	CpG	Cases No./ Controls No.	Position	BC Cases Mean±SD	Controls Mean±SD	P^a value	P^b value
IGF2	cg09694722	48/48	Body;TSS1500;5'UTR	0.078±0.022	0.077±0.015	*0.98*	*0.66*
	cg10037494	48/48	Body;TSS1500;5'UTR	0.040±0.010	0.039±0.009	*0.99*	*0.63*
	cg10337079	48/48	Body;5'UTR	0.070±0.011	0.074±0.013	*0.85*	*0.64*
	cg10501065	48/48	Body;5'UTR	0.109±0.014	0.116±0.017	*0.29*	*0.41*
	cg10650127	48/48	TSS200;Body	0.788±0.029	0.777±0.046	*0.57*	*0.77*
	cg10659464	48/48	Body;TSS1500;5'UTR	0.151±0.020	0.154±0.024	*0.93*	*1.00*
	cg11005826	48/48	Body;5'UTR	0.099±0.028	0.097±0.023	*0.99*	*0.64*
	cg11701022	48/48	Body;5'UTR;TSS200	0.209±0.021	0.212±0.031	*0.87*	*0.90*
	cg11717189	48/48	Body;3'UTR	0.428±0.106	0.438±0.090	*0.68*	*0.63*
	cg11915650	48/48	Body;TSS1500;5'UTR	0.053±0.011	0.052±0.010	*0.95*	*0.51*
	cg12322132	48/48	Body;5'UTR	0.058±0.012	0.057±0.010	*0.84*	*0.81*
	cg12528452	48/48	Body;3'UTR	0.640±0.052	0.637±0.053	*0.75*	*0.95*
	cg12614029	48/48	Body;TSS1500;5'UTR	0.042±0.007	0.043±0.006	*0.88*	*0.95*
	cg12773325	48/48	Body;TSS1500;5'UTR	0.037±0.005	0.037±0.005	*0.89*	*0.98*
	cg12877935	48/48	Body;5'UTR	0.036±0.005	0.037±0.006	*0.37*	*0.73*
	cg13165070	48/48	Body;3'UTR	0.392±0.095	0.401±0.082	*0.68*	*0.95*
	cg13167664	48/48	Body;5'UTR	0.016±0.002	0.017±0.004	*0.96*	*0.85*
	cg13756879	48/48	Body;TSS1500;5'UTR	0.036±0.007	0.038±0.011	*0.97*	*0.99*
	cg13791131	48/48	Body;5'UTR	0.074±0.019	0.089±0.030	*0.21*	*0.29*
	cg13928782	48/48	Body;5'UTR;TSS200	0.088±0.021	0.090±0.026	*0.99*	*0.87*
	cg14188639	48/48	Body;TSS1500;5'UTR	0.106±0.033	0.111±0.048	*0.93*	*0.92*
	cg14432744	48/48	Body;TSS1500	0.575±0.035	0.573±0.037	*0.98*	*0.90*
	cg14608156	48/48	Body;TSS1500;5'UTR	0.030±0.003	0.031±0.003	*0.61*	*0.77*
	cg14890224	48/48	TSS1500;Body	0.633±0.025	0.625±0.025	*0.41*	*0.86*
	cg14895961	48/48	Body;5'UTR;TSS1500	0.274±0.066	0.266±0.065	*0.92*	*0.75*
	cg15168906	48/48	Body;5'UTR;TSS1500	0.552±0.047	0.563±0.072	*0.90*	*0.72*
	cg15393937	48/48	Body;TSS1500;5'UTR	0.155±0.045	0.154±0.031	*0.99*	*0.84*
	cg15508379	48/48	Body;5'UTR;TSS200	0.122±0.022	0.124±0.026	*0.99*	*0.81*
	cg16415340	48/48	Body;TSS1500;5'UTR	0.038±0.010	0.040±0.011	*0.76*	*0.97*
	cg16817891	48/48	Body;5'UTR	0.043±0.010	0.041±0.011	*0.79*	*0.67*
	cg16977706	48/48	Body;5'UTR;1stExon	0.097±0.021	0.102±0.020	*0.79*	*0.80*
	cg17037101	48/48	Body;5'UTR;TSS1500	0.062±0.028	0.062±0.021	*0.89*	*0.89*
	cg17300736	48/48	Body;5'UTR;TSS200	0.057±0.011	0.059±0.010	*0.63*	*0.95*
	cg17434309	48/48	Body;5'UTR;TSS1500	0.060±0.043	0.057±0.028	*0.97*	*0.78*
	cg17462140	48/48	Body;5'UTR;TSS200	0.108±0.019	0.108±0.014	*0.94*	*0.68*
	cg17665927	48/48	Body;1stExon;5'UTR	0.734±0.059	0.730±0.058	*0.91*	*0.95*
	cg18087943	48/48	Body;5'UTR;TSS1500	0.022±0.002	0.023±0.002	*0.92*	*0.84*
	cg19002337	48/48	Body;5'UTR;TSS1500	0.031±0.005	0.031±0.005	*0.97*	*0.82*

Table S4. Continued

	CpG	Cases No./ Controls No.	Position	BC Cases Mean±SD	Controls Mean±SD	P^a value	P^b value
IGF2	cg19131227	48/48	Body;5'UTR;TSS1500	0.285±0.050	0.292±0.053	*0.86*	*0.97*
	cg19137676	48/48	Body;TSS1500	0.461±0.049	0.457±0.038	*0.80*	*0.90*
	cg19371526	48/48	Body;5'UTR;TSS1500	0.096±0.019	0.093±0.014	*0.61*	*0.37*
	cg19443075	48/48	Body;5'UTR;TSS1500	0.180±0.033	0.178±0.026	*0.95*	*0.72*
	cg19642877	48/48	Body;3'UTR;5'UTR	0.522±0.035	0.524±0.024	*0.93*	*0.93*
	cg20088847	48/48	Body;5'UTR;TSS1500	0.072±0.010	0.079±0.031	*0.26*	*0.56*
	cg20339650	48/48	Body;5'UTR;TSS1500	0.146±0.021	0.147±0.018	*0.99*	*0.82*
	cg20728696	48/48	Body;5'UTR	0.071±0.014	0.073±0.011	*0.66*	*0.77*
	cg20766090	48/48	Body;5'UTR	0.025±0.003	0.025±0.003	*0.82*	*0.93*
	cg20792294	48/48	Body;5'UTR	0.028±0.009	0.028±0.008	*0.90*	*0.74*
	cg20895511	48/48	Body;5'UTR;1stExon	0.186±0.033	0.185±0.030	*0.86*	*0.89*
	cg21237591	48/48	Body;5'UTR;TSS200	0.155±0.033	0.158±0.029	*0.79*	*0.98*
	cg21532432	48/48	Body;5'UTR	0.065±0.015	0.071±0.015	*0.42*	*0.74*
	cg21667878	48/48	Body;5'UTR;TSS1500	0.038±0.013	0.038±0.011	*0.93*	*0.77*
	cg21728792	48/48	TSS1500;Body	0.914±0.017	0.915±0.012	*0.97*	*0.96*
	cg22225943	48/48	Body;5'UTR;TSS200	0.158±0.035	0.165±0.034	*0.67*	*0.94*
	cg22287492	48/48	Body;5'UTR;TSS1500	0.162±0.040	0.165±0.030	*0.93*	*0.94*
	cg22932993	48/48	TSS1500;Body	0.912±0.011	0.914±0.010	*0.75*	*0.94*
	cg22956483	48/48	Body	0.304±0.033	0.303±0.026	*0.90*	*0.95*
	cg23030069	48/48	Body;5'UTR;1stExon	0.040±0.007	0.040±0.006	*0.91*	*0.49*
	cg23676551	48/48	Body;5'UTR;TSS1500	0.119±0.031	0.124±0.026	*0.84*	*0.86*
	cg23889607	48/48	Body;3'UTR	0.962±0.009	0.963±0.006	*0.72*	*0.62*
	cg23905216	48/48	Body;TSS1500;5'UTR	0.170±0.029	0.172±0.036	*0.99*	*0.98*
	cg24047810	48/48	Body;5'UTR;TSS200	0.210±0.025	0.219±0.028	*0.56*	*0.99*
	cg24183187	48/48	TSS1500;Body	0.878±0.012	0.880±0.012	*0.50*	*0.62*
	cg24366657	48/48	Body;TSS1500;5'UTR	0.045±0.011	0.048±0.017	*0.85*	*0.95*
	cg24431667	48/48	Body;5'UTR;TSS200	0.059±0.011	0.059±0.010	*0.89*	*0.53*
	cg24439505	48/48	Body;TSS1500	0.637±0.058	0.634±0.057	*0.93*	*0.95*
	cg24781163	48/48	Body;5'UTR;TSS1500	0.533±0.074	0.545±0.062	*0.77*	*0.78*
	cg24917382	48/48	Body;TSS1500;5'UTR	0.133±0.022	0.136±0.023	*0.99*	*0.97*
	cg25163476	48/48	Body;5'UTR;TSS200;TSS1500	0.086±0.039	0.083±0.012	*1.00*	*0.92*
	cg25574024	48/48	Body;5'UTR	0.070±0.020	0.078±0.024	*0.58*	*0.68*
	cg25742037	48/48	Body;TSS1500	0.423±0.053	0.411±0.060	*0.69*	*0.38*
	cg25763864	48/48	Body;3'UTR	0.924±0.010	0.925±0.010	*0.99*	*0.80*
	cg26401390	48/48	Body	0.494±0.041	0.499±0.034	*0.80*	*0.73*
	cg26517849	48/48	Body;TSS1500;5'UTR	0.031±0.003	0.032±0.003	*0.79*	*0.74*
	cg26719629	48/48	Body;5'UTR;TSS1500	0.340±0.108	0.339±0.070	*0.98*	*0.90*
	cg26913576	48/48	Body;3'UTR	0.892±0.017	0.893±0.015	*0.64*	*0.92*
	cg27263998	48/48	TSS200;Body	0.544±0.050	0.541±0.041	*0.95*	*0.95*
	cg27331871	48/48	Body;TSS1500	0.238±0.038	0.236±0.068	*0.98*	*0.80*

Table S4. Continued

	Author, year	Cases No./ Controls No.	Meth (BC cases)	Meth (controls)	*P* value	CpG	Cases No./ Controls No.	Position	BC Cases Mean±SD	Controls Mean±SD	P^a value	P^b value
SYK	Zmetakova I, 2013*[10]	34/50	1.15 ± 0.44	1.06 ± 0.24	*0.638*	cg02608019	48/48	TSS200;5'UTR;1stExon	0.031±0.004	0.031±0.004	*0.84*	*0.89*
	Widschwendter M, 2008[23]	320/676	2.2	2.4	*0.889*	cg05801648	48/48	Body;5'UTR	0.036±0.004	0.034±0.004	*0.92*	*0.28*
						cg07160163	48/48	TSS1500	0.034±0.008	0.035±0.008	*0.85*	*0.96*
						cg10025443	48/48	Body;5'UTR	0.029±0.007	0.029±0.009	*0.87*	*0.92*
						cg13782919	48/48	Body;5'UTR;TSS1500	0.673±0.036	0.665±0.033	*0.64*	*0.58*
						cg13897882	48/48	Body;3'UTR	0.920±0.015	0.919±0.012	*0.90*	*0.70*
						cg14005120	48/48	Body	0.894±0.026	0.894±0.019	*0.96*	*0.42*
						cg14054883	48/48	Body	0.798±0.059	0.768±0.061	*0.12*	*0.86*
						cg14055502	48/48	Body;3'UTR	0.913±0.018	0.910±0.016	*0.71*	*0.62*
						cg14304761	48/48	TSS200;5'UTR;1stExon	0.022±0.005	0.021±0.002	*0.98*	*0.73*
						cg14424519	48/48	Body	0.903±0.010	0.903±0.011	*0.90*	*0.80*
						cg16896647	48/48	TSS1500	0.050±0.012	0.052±0.011	*0.90*	*0.87*
						cg23447996	48/48	Body;5'UTR	0.064±0.016	0.068±0.014	*0.85*	*0.87*

*Zmetakova I, 2013. DNA methylaton was investigated between breast cancer patients and controls in peripheral blood cells.

‡Zmetakova I, 2013. DNA methylaton was investigated between breast cancer patients and controls in plasma samples.

† literature based on review by Tang *et al.* 2016[28].

P^a value was adjusted by age, batch and multiple test.

P^b value was adjusted by age, batch, cell counts and multiple test.

Significant *P* values are in bold, α= 0.05.

Abbreviations: BC: breast cancer.

Figure S1. Location of CpG sites in gene promoter region

RASSF1A

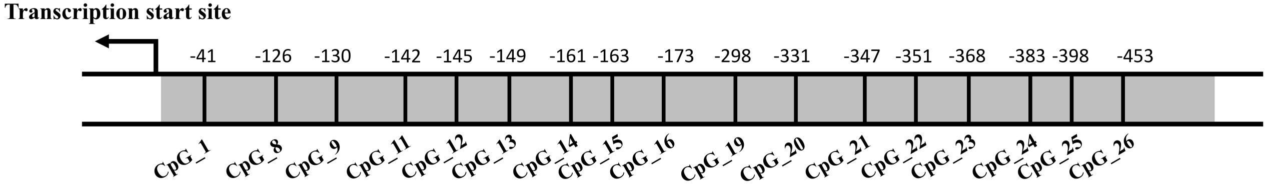

ATM

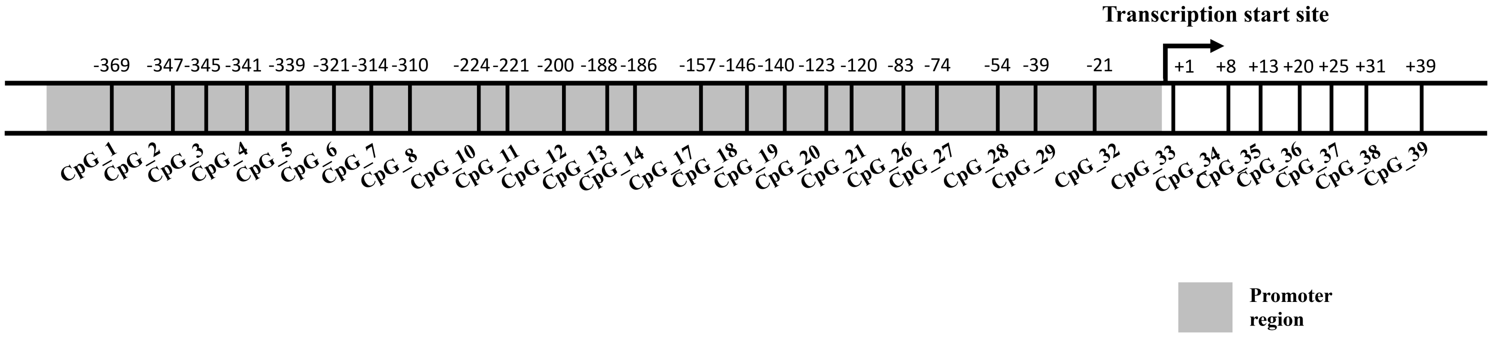

Figure S1. The schematic graph shows the location of CpG sites in the promoter region of *RASSF1A* and *ATM*.

References

1. Cho, Y. H.; McCullough, L. E.; Gammon, M. D.; Wu, H. C.; Zhang, Y. J.; Wang, Q.; Xu, X.; Teitelbaum, S. L.; Neugut, A. I.; Chen, J.; Santella, R. M., Promoter Hypermethylation in White Blood Cell DNA and Breast Cancer Risk. J Cancer 2015, 6, (9), 819-24.
2. Gupta, S.; Jaworska-Bieniek, K.; Narod, S. A.; Lubinski, J.; Wojdacz, T. K.; Jakubowska, A., Methylation of the BRCA1 promoter in peripheral blood DNA is associated with triple-negative and medullary breast cancer. Breast Cancer Res Treat 2014, 148, (3), 615-22.
3. Bosviel, R.; Garcia, S.; Lavediaux, G.; Michard, E.; Dravers, M.; Kwiatkowski, F.; Bignon, Y. J.; Bernard-Gallon, D. J., BRCA1 promoter methylation in peripheral blood DNA was identified in sporadic breast cancer and controls. Cancer Epidemiol 2012, 36, (3), e177-82.
4. Wong, E. M.; Southey, M. C.; Fox, S. B.; Brown, M. A.; Dowty, J. G.; Jenkins, M. A.; Giles, G. G.; Hopper, J. L.; Dobrovic, A., Constitutional methylation of the BRCA1 promoter is specifically associated with BRCA1 mutation-associated pathology in early-onset breast cancer. Cancer Prev Res (Phila) 2011, 4, (1), 23-33.
5. Iwamoto, T.; Yamamoto, N.; Taguchi, T.; Tamaki, Y.; Noguchi, S., BRCA1 promoter methylation in peripheral blood cells is associated with increased risk of breast cancer with BRCA1 promoter methylation. Breast Cancer Res Treat 2011, 129, (1), 69-77.
6. Snell, C.; Krypuy, M.; Wong, E. M.; Loughrey, M. B.; Dobrovic, A., BRCA1 promoter methylation in peripheral blood DNA of mutation negative familial breast cancer patients with a BRCA1 tumour phenotype. Breast Cancer Res 2008, 10, (1), R12.
7. Cho, Y. H.; Yazici, H.; Wu, H. C.; Terry, M. B.; Gonzalez, K.; Qu, M.; Dalay, N.; Santella, R. M., Aberrant promoter hypermethylation and genomic hypomethylation in tumor, adjacent normal tissues and blood from breast cancer patients. Anticancer Res 2010, 30, (7), 2489-96.
8. Radpour, R.; Barekati, Z.; Kohler, C.; Lv, Q.; Burki, N.; Diesch, C.; Bitzer, J.; Zheng, H.; Schmid, S.; Zhong, X. Y., Hypermethylation of tumor suppressor genes involved in critical regulatory pathways for developing a blood-based test in breast cancer. PLoS One 2011, 6, (1), e16080.
9. Liu, L.; Sun, L.; Li, C.; Li, X.; Zhang, Y.; Yu, Y.; Xia, W., Quantitative detection of methylation of FHIT and BRCA1 promoters in the serum of ductal breast cancer patients. Biomed Mater Eng 2015, 26 Suppl 1, S2217-22.
10. Zmetakova, I.; Danihel, L.; Smolkova, B.; Mego, M.; Kajabova, V.; Krivulcik, T.; Rusnak, I.; Rychly, B.; Danis, D.; Repiska, V.; Blasko, P.; Karaba, M.; Benca, J.; Pechan, J.; Fridrichova, I., Evaluation of protein expression and DNA methylation profiles detected by pyrosequencing in invasive breast cancer. Neoplasma 2013, 60, (6), 635-46.
11. Kloten, V.; Becker, B.; Winner, K.; Schrauder, M. G.; Fasching, P. A.; Anzeneder, T.; Veeck, J.; Hartmann, A.; Knuchel, R.; Dahl, E., Promoter hypermethylation of the tumor-suppressor genes ITIH5, DKK3, and RASSF1A as novel biomarkers for blood-based breast cancer screening. Breast Cancer Res 2013, 15, (1), R4.
12. Ahmed, I. A.; Pusch, C. M.; Hamed, T.; Rashad, H.; Idris, A.; El-Fadle, A. A.; Blin, N., Epigenetic alterations by methylation of RASSF1A and DAPK1 promoter sequences in mammary carcinoma detected in extracellular tumor DNA. Cancer Genet Cytogenet 2010, 199, (2), 96-100.
13. Brooks, J. D.; Cairns, P.; Shore, R. E.; Klein, C. B.; Wirgin, I.; Afanasyeva, Y.; Zeleniuch-Jacquotte, A., DNA methylation in pre-diagnostic serum samples of breast cancer cases: results of a nested case-control study. Cancer Epidemiol 2010, 34, (6), 717-23.
14. Kim, J. H.; Shin, M. H.; Kweon, S. S.; Park, M. H.; Yoon, J. H.; Lee, J. S.; Choi, C.; Fackler, M. J.; Sukumar, S., Evaluation of promoter hypermethylation detection in serum as a diagnostic tool for breast carcinoma in Korean women. Gynecol Oncol 2010, 118, (2), 176-81.
15. Yazici, H.; Terry, M. B.; Cho, Y. H.; Senie, R. T.; Liao, Y.; Andrulis, I.; Santella, R. M., Aberrant methylation of RASSF1A in plasma DNA before breast cancer diagnosis in the Breast Cancer Family Registry. Cancer Epidemiol Biomarkers Prev 2009, 18, (10), 2723-5.
16. Hoque, M. O.; Feng, Q.; Toure, P.; Dem, A.; Critchlow, C. W.; Hawes, S. E.; Wood, T.; Jeronimo, C.; Rosenbaum, E.; Stern, J.; Yu, M.; Trink, B.; Kiviat, N. B.; Sidransky, D., Detection of aberrant methylation of four genes in plasma DNA for the detection of breast cancer. J Clin Oncol 2006, 24, (26), 4262-9.
17. Van der Auwera, I.; Elst, H. J.; Van Laere, S. J.; Maes, H.; Huget, P.; van Dam, P.; Van Marck, E. A.; Vermeulen, P. B.; Dirix, L. Y., The presence of circulating total DNA and methylated genes is associated with circulating tumour cells in blood from breast cancer patients. Br J Cancer 2009, 100, (8), 1277-86.

18. Papadopoulou, E.; Davilas, E.; Sotiriou, V.; Georgakopoulos, E.; Georgakopoulou, S.; Koliopanos, A.; Aggelakis, F.; Dardoufas, K.; Agnanti, N. J.; Karydas, I.; Nasioulas, G., Cell-free DNA and RNA in plasma as a new molecular marker for prostate and breast cancer. Ann N Y Acad Sci 2006, 1075, 235-43.
19. Dulaimi, E.; Hillinck, J.; Ibanez de Caceres, I.; Al-Saleem, T.; Cairns, P., Tumor suppressor gene promoter hypermethylation in serum of breast cancer patients. Clin Cancer Res 2004, 10, (18 Pt 1), 6189-93.
20. Swellam, M.; Abdelmaksoud, M. D.; Sayed Mahmoud, M.; Ramadan, A.; Abdel-Moneem, W.; Hefny, M. M., Aberrant methylation of APC and RARbeta2 genes in breast cancer patients. IUBMB Life 2015, 67, (1), 61-8.
21. Brennan, K.; Garcia-Closas, M.; Orr, N.; Fletcher, O.; Jones, M.; Ashworth, A.; Swerdlow, A.; Thorne, H.; Riboli, E.; Vineis, P.; Dorronsoro, M.; Clavel-Chapelon, F.; Panico, S.; Onland-Moret, N. C.; Trichopoulos, D.; Kaaks, R.; Khaw, K. T.; Brown, R.; Flanagan, J. M., Intragenic ATM methylation in peripheral blood DNA as a biomarker of breast cancer risk. Cancer Res 2012, 72, (9), 2304-13.
22. Flanagan, J. M.; Munoz-Alegre, M.; Henderson, S.; Tang, T.; Sun, P.; Johnson, N.; Fletcher, O.; Dos Santos Silva, I.; Peto, J.; Boshoff, C.; Narod, S.; Petronis, A., Gene-body hypermethylation of ATM in peripheral blood DNA of bilateral breast cancer patients. Hum Mol Genet 2009, 18, (7), 1332-42.
23. Widschwendter, M.; Apostolidou, S.; Raum, E.; Rothenbacher, D.; Fiegl, H.; Menon, U.; Stegmaier, C.; Jacobs, I. J.; Brenner, H., Epigenotyping in peripheral blood cell DNA and breast cancer risk: a proof of principle study. PLoS One 2008, 3, (7), e2656.
24. Zurita, M.; Lara, P. C.; del Moral, R.; Torres, B.; Linares-Fernandez, J. L.; Arrabal, S. R.; Martinez-Galan, J.; Oliver, F. J.; Ruiz de Almodovar, J. M., Hypermethylated 14-3-3-sigma and ESR1 gene promoters in serum as candidate biomarkers for the diagnosis and treatment efficacy of breast cancer metastasis. BMC Cancer 2010, 10, 217.
25. Martinez-Galan, J.; Torres, B.; Del Moral, R.; Munoz-Gamez, J. A.; Martin-Oliva, D.; Villalobos, M.; Nunez, M. I.; Luna Jde, D.; Oliver, F. J.; Ruiz de Almodovar, J. M., Quantitative detection of methylated ESR1 and 14-3-3-sigma gene promoters in serum as candidate biomarkers for diagnosis of breast cancer and evaluation of treatment efficacy. Cancer Biol Ther 2008, 7, (6), 958-65.
26. Harrison, K.; Hoad, G.; Scott, P.; Simpson, L.; Horgan, G. W.; Smyth, E.; Heys, S. D.; Haggarty, P., Breast cancer risk and imprinting methylation in blood. Clin Epigenetics 2015, 7, 92.
27. Ito, Y.; Koessler, T.; Ibrahim, A. E.; Rai, S.; Vowler, S. L.; Abu-Amero, S.; Silva, A. L.; Maia, A. T.; Huddleston, J. E.; Uribe-Lewis, S.; Woodfine, K.; Jagodic, M.; Nativio, R.; Dunning, A.; Moore, G.; Klenova, E.; Bingham, S.; Pharoah, P. D.; Brenton, J. D.; Beck, S.; Sandhu, M. S.; Murrell, A., Somatically acquired hypomethylation of IGF2 in breast and colorectal cancer. Hum Mol Genet 2008, 17, (17), 2633-43.
28. Tang, Q.; Cheng, J.; Cao, X.; Surowy, H.; Burwinkel, B., Blood-based DNA methylation as biomarker for breast cancer: a systematic review. Clin Epigenetics 2016, 8, 115.
